# TMEM16E regulates endothelial cell procoagulant activity and thrombosis

**DOI:** 10.1172/JCI163808

**Published:** 2023-06-01

**Authors:** Alec A. Schmaier, Papa F. Anderson, Siyu M. Chen, Emale El-Darzi, Ivan Aivasovsky, Milan P. Kaushik, Kelsey D. Sack, H. Criss Hartzell, Samir M. Parikh, Robert Flaumenhaft, Sol Schulman

**Affiliations:** 1 Division of Cardiovascular Medicine and; 2 Division of Hemostasis and Thrombosis, Beth Israel Deaconess Medical Center and Harvard Medical School, Boston, Massachusetts, USA.; 3 Cardiovascular Research Center,; 4 Department of Medicine, and; 5 Division of Pulmonary, Critical Care and Sleep Medicine, Beth Israel Deaconess Medical Center, Boston, Massachusetts, USA.; 6 Department of Cell Biology, Emory University School of Medicine, Atlanta, Georgia, USA.; 7 Division of Nephrology, Beth Israel Deaconess Medical Center and Harvard Medical School, Boston, Massachusetts, USA.; 8 Division of Nephrology and Departments of Internal Medicine and Pharmacology, University of Texas Southwestern Medical School, Dallas, Texas, USA.; 9 Division of Hematology and Hematologic Malignancies, Beth Israel Deaconess Medical Center and Harvard Medical School, Boston, Massachusetts, USA.

**Keywords:** Hematology, Vascular Biology, Endothelial cells, Thrombosis

## Abstract

Endothelial cells (ECs) normally form an anticoagulant surface under physiological conditions, but switch to support coagulation following pathogenic stimuli. This switch promotes thrombotic cardiovascular disease. To generate thrombin at physiologic rates, coagulation proteins assemble on a membrane containing anionic phospholipid, most notably phosphatidylserine (PS). PS can be rapidly externalized to the outer cell membrane leaflet by phospholipid “scramblases,” such as TMEM16F. TMEM16F-dependent PS externalization is well characterized in platelets. In contrast, how ECs externalize phospholipids to support coagulation is not understood. We employed a focused genetic screen to evaluate the contribution of transmembrane phospholipid transport on EC procoagulant activity. We identified 2 TMEM16 family members, TMEM16F and its closest paralog, TMEM16E, which were both required to support coagulation on ECs via PS externalization. Applying an intravital laser-injury model of thrombosis, we observed, unexpectedly, that PS externalization was concentrated at the vessel wall, not on platelets. TMEM16E-null mice demonstrated reduced vessel-wall–dependent fibrin formation. The TMEM16 inhibitor benzbromarone prevented PS externalization and EC procoagulant activity and protected mice from thrombosis without increasing bleeding following tail transection. These findings indicate the activated endothelial surface is a source of procoagulant phospholipid contributing to thrombus formation. TMEM16 phospholipid scramblases may be a therapeutic target for thrombotic cardiovascular disease.

## Graphical abstract

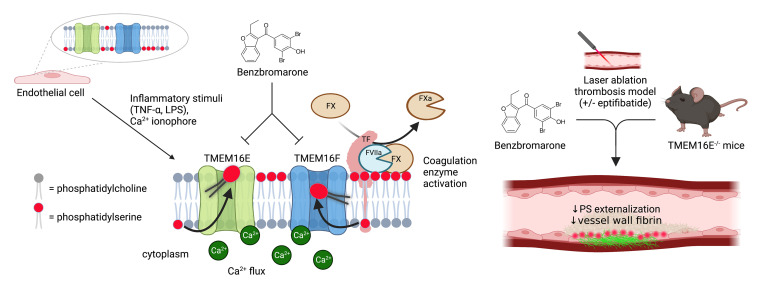

## Introduction

Thrombotic disorders such as myocardial infarction, stroke, and venous thromboembolism are leading causes of mortality worldwide (1). Blood coagulation, and therefore thrombosis, depends at key stages on a membrane surface containing anionic phospholipid, most commonly phosphatidylserine (PS) (2). PS promotes the recruitment and activation of coagulation factor X by the tissue factor–factor VIIa (TF-VIIa) complex (3, 4) and assembly and activity of the factor Xa-Va-prothrombinase coagulation enzyme complex (5, 6), the initiating and ultimate steps in thrombin formation, respectively. Coagulation factor interaction with PS accelerates enzyme kinetics by at least 3 orders of magnitude to physiologic rates (6, 7). PS constitutes approximately 10%–15% of plasma membrane phospholipid (8), but under basal conditions is sequestered on the inner membrane leaflet and is therefore inaccessible to the extracellular environment. A sustained rise in intracellular calcium (Ca^2+^) triggers PS externalization via Ca^2+^-activated phospholipid scramblases (PLSs), transmembrane channels that allow PS to move down its concentration gradient to the outer leaflet of the plasma membrane (9–11). By preventing enzyme complex assembly (12, 13), PS-binding proteins such as annexin V and lactadherin inhibit coagulation reactions in vitro (14, 15) and decrease thrombosis in vivo (15–17). These findings suggest that PS exposure is an integral step in thrombosis and that targeting PS may be a viable antithrombotic strategy.

Following agonist stimulation in vitro, activated platelets readily externalize PS and support thrombin generation, providing an accessible means of studying cell-based PS exposure in coagulation (7, 10). It has therefore been suggested that activated platelets are the major source of procoagulant PS in vivo and thus the major site of coagulation enzyme-complex assembly for hemostasis and thrombosis (18). The identification of TMEM16F as a PLS required for PS externalization in platelets (19, 20) and the finding that mutations in TMEM16F underlie the mild-to-moderate bleeding disorder Scott syndrome (19, 21) led to further investigation of TMEM16F in platelet function. Platelets require TMEM16F to externalize PS in response to stimuli that raise intracellular Ca^2+^ (22). Loss of phospholipid scrambling in platelets protects from thrombosis or impairs hemostasis in some studies but not in others (22–25). These findings would suggest that sources of PS beyond the platelet and additional proteins beyond TMEM16F may regulate procoagulant phospholipid externalization.

Endothelial cells (ECs) form a constitutive anticoagulant surface under basal conditions to maintain blood flow. Loss of this anticoagulant property is considered a hallmark of cardiovascular disease, leading to thrombosis. However, the relative contribution of activated endothelium to blood clotting in vivo is not well understood. Several pathologic stimuli, including hypoxia, cytokines, and lipopolysaccharide, induce PS externalization on ECs (26–28), but the physiologic significance of EC phospholipid scrambling and the proteins that regulate it remain poorly characterized, although TMEM16F is likely involved (29). Inflammatory stimuli also promote TF expression and its procoagulant activity in ECs (30, 31). The majority of cell surface–expressed TF binds factor VII/VIIa, but exists in a deactivated state (32, 33). PS externalization is at least partly responsible for enhancing the TF-dependent catalytic activity of factor VIIa (3, 4, 34, 35). Therefore, identification of regulators of PS externalization will aid our understanding of TF activation and inflammatory thrombosis.

To identify regulators of membrane procoagulant activity in ECs, we performed a targeted screen of genes encoding proteins predicted to regulate transmembrane phospholipid transport for their contribution to factor VIIa–catalyzed activation of factor X. Using this approach, we identified 2 TMEM16 family members, TMEM16F and its closest paralog, TMEM16E (36), that support EC procoagulant activity via PS externalization. The TMEM16 family comprises Ca^2+^-activated transmembrane proteins that function as ion channels, PLSs, or, in some cases, both (36). TMEM16E is highly expressed in skeletal muscle, where it regulates muscle regeneration and repair (37, 38), but is not expressed in platelets (22, 39, 40) and has no previously known role in hemostasis or thrombosis. Our findings from intravital thrombosis assays suggest that the vessel wall is a major source of PS during thrombus formation. We demonstrate that both genetic deletion of TMEM16E and pharmacologic blockade of TMEM16 proteins inhibit fibrin formation in a vessel-wall–dependent manner during thrombosis in vivo. These results suggest that endothelial-derived PS contributes to thrombus formation and that small-molecule inhibition of TMEM16 may be a novel antithrombotic strategy.

## Results

### Identification of transmembrane phospholipid transporters predicted to regulate EC procoagulant activity.

**Figure 1:**
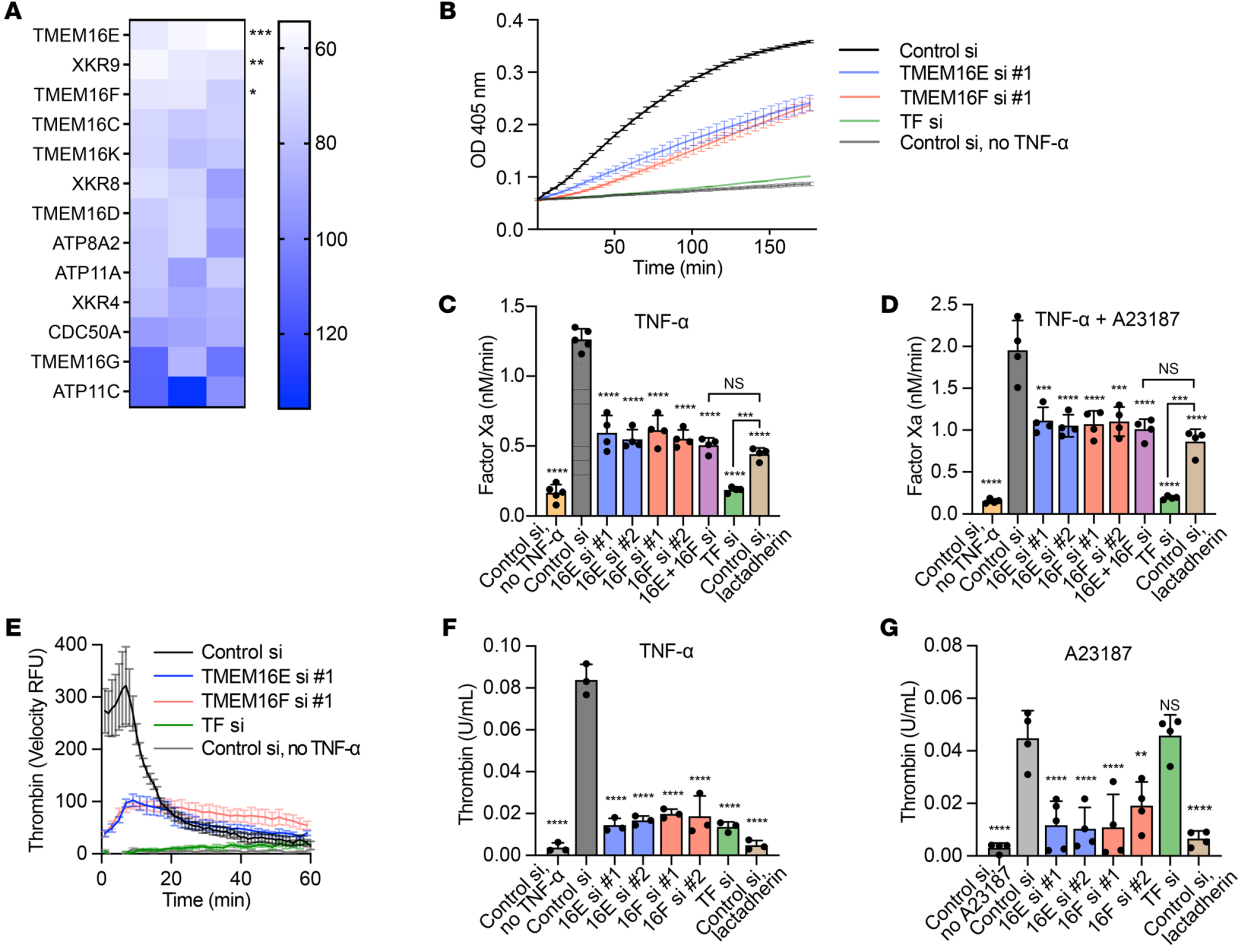
TMEM16E and TMEM16F regulate EC procoagulant activity. (**A**) Heatmap illustrating the relative positive or negative regulation of factor VIIa–catalyzed activation of factor X following silencing of the indicated genes in HUVECs. Indicated genes were silenced with a pool of 4 distinct siRNAs and tested in triplicate. Each box represents an independent experimental plate, with scale bar depicting percentage of factor Xa generated compared with cells transfected with untargeted control siRNA. Lighter color indicates lower percentage factor Xa generation compared with control. (**B**–**G**) HUVECs were transfected with individual siRNAs for 72 hours and assayed for their ability to support factor VIIa–catalyzed activation of factor X (**B**–**D**) or thrombin generation in plasma-treated ECs (**E**–**G**). Cells were stimulated with TNF-α (10 ng/mL) for 3.5 hours (**B**, **C**, **E**, and **F**), TNF-α for 3.5 hours plus Ca^2+^ ionophore A23187 (6 μM) for 20 minutes (**D**), or A23187 alone for 20 minutes (**G**). Representative experiments are depicted as mean absorbance for factor Xa generation (**B**) or the first derivative of arbitrary fluorescent units for thrombin generation (**E**) as a function of time. 16E, 16F, and TF denote siRNA targeting TMEM16E, TMEM16F, and TF, respectively, and #1 and #2 denote distinct siRNA sequences. When indicated, lactadherin (100 nM) was added to cells treated with control siRNA. *n* = 3–5 independent experiments. Error bars indicate mean ± SEM (**B** and **E**) or mean ± SD (**C**, **D**, **F**, and **G**). Asterisks denoting significance are in reference to control siRNA, unless otherwise specified with brackets to indicate pairwise comparison, ANOVA with Tukey’s post test. *P < 0.05; **P < 0.01; ***P < 0.001; ****P < 0.0001.

We used siRNA to evaluate genes implicated in regulation of membrane phospholipid asymmetry and measured coagulation initiation on TNF-α–stimulated ECs. Primary human umbilical vein ECs (HUVECs) were transfected with gene-specific pools of 4 distinct siRNAs and cultured for 72 hours prior to TNF-α stimulation. The cells were then assayed for their ability to support factor VIIa–catalyzed conversion of factor X to factor Xa in a chromogenic assay (Figure 1A). Since membrane PS composition is a critical determinant of factor X activation by the TF-VIIa complex (3, 34, 35), our approach was able to identify regulators of transmembrane phospholipid exchange that influenced coagulation. We focused on 13 validated genes known to affect the outer leaflet expression of PS in biological membranes, including 6 members of the TMEM16 family of Ca^2+^-activated PLSs, 3 members of the Xk-related family of caspase-activated PLSs (41), and 3 members of the P4-ATPase family of phospholipid flippases (42), including their cofactor, CDC50A. We identified TMEM16E, Xkr9, and TMEM16F as significant regulators of factor VIIa–catalyzed activation of factor X in ECs (*P* = 0.0004, 0.0016, and 0.0113, respectively, as compared with control siRNA; Figure 1A). TMEM16E is the closest paralog of TMEM16F, which is the canonical Ca^2+^-activated PLS (36). Both TMEM16E and TMEM16F have been shown to have PLS activity (37,43). Primary human ECs from multiple tissues expressed both TMEM16E and TMEM16F in a manner independent of TNF-α stimulation (Supplemental Figure 1; supplemental material available online with this article; https://doi.org/10.1172/JCI163808DS1). In contrast, we could not verify gene expression of *XKR9* in multiple types of ECs using quantitative PCR (qPCR). Therefore, we focused our attention on TMEM16E and TMEM16F as PLSs that may promote coagulation on ECs.

### TMEM16E and TMEM16F are required for procoagulant activity in ECs.

To corroborate these findings, we tested the requirement of TMEM16E and TMEM16F in supporting coagulation on ECs. We validated 2 distinct siRNAs for their ability to inhibit expression of TMEM16E or TMEM16F in ECs (Supplemental Figure 2). We silenced TMEM16E or TMEM16F in primary HUVECs with these individual siRNAs and stimulated cells with TNF-α to induce expression of TF. Cells were then tested for their ability to support factor VIIa–catalyzed activation of factor X in a kinetic factor Xa generation assay. Silencing of either TMEM16E or TMEM16F resulted in approximately 50% reduction of factor Xa generation compared with addition of nontargeting control siRNA (Figure 1, B and C). Silencing of TMEM16E or TMEM16F also inhibited factor VIIa–catalyzed activation of factor X that was augmented by addition of ionophore A23187 (Figure 1D), which promotes PS externalization by raising intracellular Ca^2+^ (9, 10). In both cases, the degree of inhibition following silencing of TMEM16E and TMEM16F was similar to that observed following treatment with lactadherin, which neutralizes externalized PS, suggesting that TMEM16E and TMEM16F may promote procoagulant activity through PS externalization (Figure 1, C and D). Dual silencing of TMEM16E and TMEM16F did not further suppress factor Xa generation beyond silencing of either gene product alone (Figure 1, C and D; see Discussion). We observed a similar dependence on both TMEM16E and TMEM16F for factor VIIa–catalyzed activation of factor X following stimulation with lipopolysaccharide (Supplemental Figure 3, A and B). To uncouple the effects of TNF-α on TF expression and PS externalization, we used an Ea.hy926 cell line stably expressing TF (28). In Ea.hy926-TF cells, TMEM16E and TMEM16F were required for Ca^2+^ ionophore–induced augmentation of factor VIIa–catalyzed activation of factor X (Supplemental Figure 3C).

ECs stimulated with TNF-α also required TMEM16E and TMEM16F to support thrombin generation in human plasma (Figure 1, E and F). In this assay, EC-mediated thrombin generation was completely abolished by lactadherin (Figure 1F). Treatment with Ca^2+^ ionophore A23187 alone promoted thrombin generation as well and was inhibited following silencing of TMEM16E or TMEM16F or addition of lactadherin (Figure 1G). Together, these results demonstrate that both TMEM16E and TMEM16F were necessary to support maximal procoagulant activity on the EC surface.

### TMEM16E and TMEM16F regulate PS externalization on ECs.

**Figure 2:**
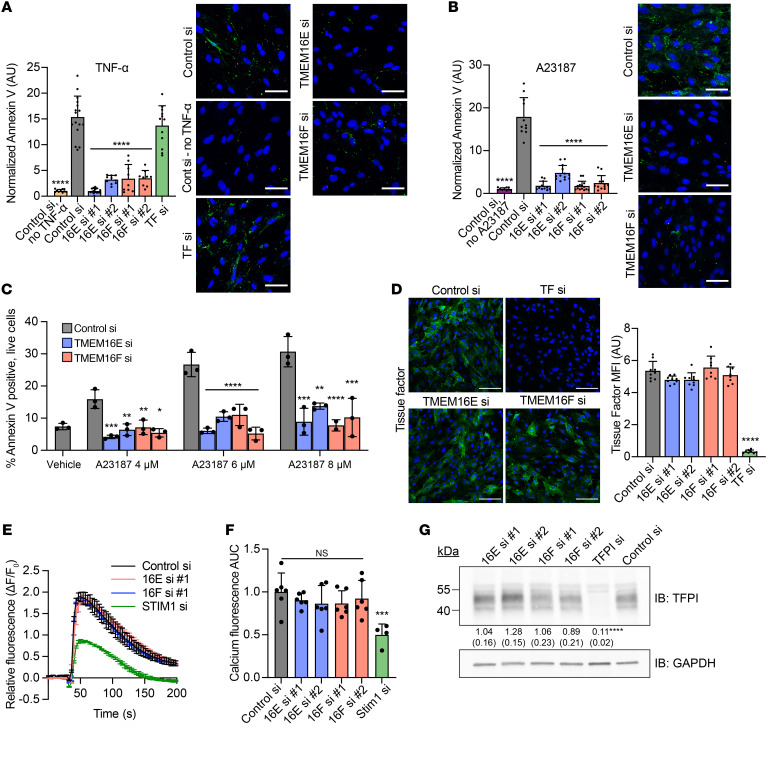
TMEM16E and TMEM16F are required for PS externalization on ECs. HUVECs were transfected with indicated siRNAs for 72 hours, stimulated with TNF-α (10 ng/mL) for 16 hours (**A**) or Ca^2+^ ionophore A23187 (6 μM) for 20 minutes (**B**), and stained with annexin V (green) to detect PS externalization and Zombie Red (red) to detect cell death. Total annexin V fluorescence was normalized to number of nuclei (blue) and dead cells. (**C**) PS externalization following treatment with ionophore A23187 was detected using annexin V by flow cytometry. Histograms were generated after gating on live (DAPI negative) cells only. (**D**) HUVECs were transfected with indicated siRNAs for 72 hours, stimulated with TNF-α (10 ng/mL) for 3.5 hours, and stained for TF (green). Mean fluorescent intensity (MFI) was normalized to background for each image. Representative images are shown. (**E** and **F**) Intracellular Ca^2+^ flux was measured with Calbryte 520 AM in siRNA-transfected HUVECs following stimulation with thrombin (1 U/mL). Silencing of the store-operated Ca^2+^ regulator STIM1 served as a positive control. Time course of Calbryte 520 fluorescence after thrombin stimulation normalized to background fluorescence (**E**) and AUC values (**F**) normalized to cells treated with control siRNA are shown. (**G**) TFPI protein was determined by SDS-PAGE and immunoblotting with anti-TFPI antibody in HUVECs transfected with indicated siRNAs. Numbers refer to fold-change normalized to GAPDH (± SD). Scale bars: 100 μm (**A** and **B**); 50 μm (**D**). *n* = 3–6 independent experiments. Error bars indicate mean ± SD (**A**–**D** and **F**) or mean ± SEM (**E**). Asterisks denoting significance are in reference to control siRNA, ANOVA with Tukey’s post test. *P < 0.05; **P < 0.01; ***P < 0.001; ****P < 0.0001.

TMEM16E and TMEM16F function as Ca^2+^-activated PLSs, disrupting membrane phospholipid asymmetry by allowing PS and other anionic phospholipids to move down their concentration gradients from the inner to the outer membrane leaflet (11, 37, 44). Indeed, PS externalization in response to TNF-α or Ca^2+^ ionophore was markedly inhibited in HUVECs following silencing of TMEM16E or TMEM16F, as detected by annexin V binding and immunofluorescence microscopy (Figure 2, A and B) or flow cytometry (Figure 2C). PS externalization is a common end point of apoptosis and other cell death pathways, but we did not observe an increase in dead cells after exposure to TNF-α and ionophore at the concentrations used in this study (Supplemental Figure 4). Therefore, PS externalization in these experiments was not due to cell death. Silencing of TMEM16E or TMEM16F had no effect on TNF-α–induced expression of TF on the cell surface (Figure 2D). TMEM16 proteins can also function as ion channels and have been implicated in regulating intracellular Ca^2+^ flux in response to G protein–coupled receptor signaling (20, 44, 45). Therefore, TMEM16E or TMEM16F could affect PS externalization indirectly through regulation of Ca^2+^ transients. To test this possibility, we measured intracellular Ca^2+^ following stimulation with thrombin, which induces rapid Ca^2+^ elevation in ECs. Silencing of TMEM16E or TMEM16F did not significantly affect intracellular Ca^2+^ flux (Figure 2, E and F). Although endothelial TF activity is negatively regulated by the TF pathway inhibitor (TFPI), silencing of TMEM16E or TMEM16F did not significantly affect the expression of TFPI (Figure 2G; see supplemental material for full, uncut gels). These results indicate that TMEM16E and TMEM16F regulate EC procoagulant activity via PS externalization.

### PS externalization during thrombotic injury occurs on the vessel wall.

**Figure 3:**
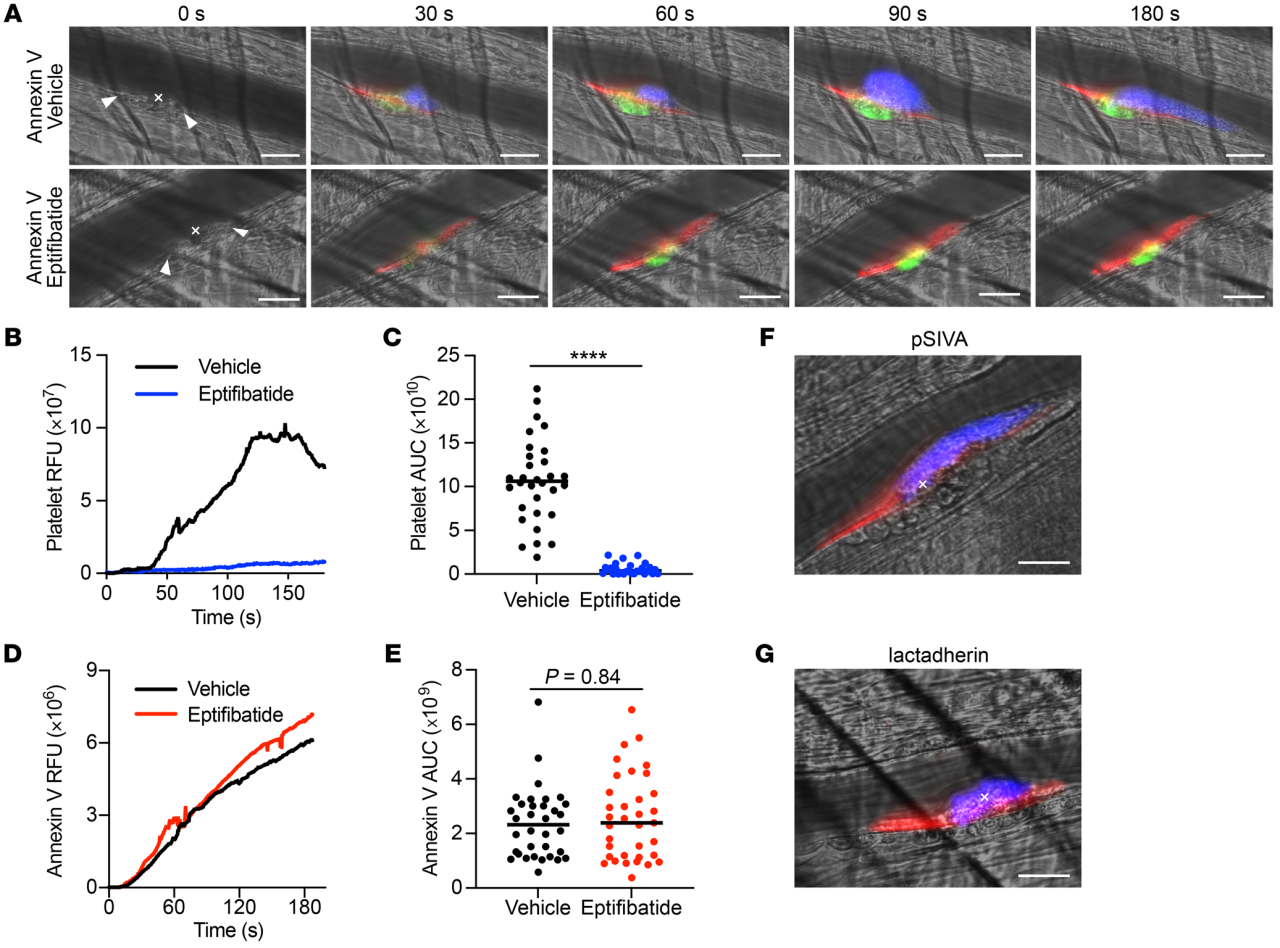
PS externalization visualized via intravital microscopy occurs on the vessel wall and is unaffected by platelet inhibition. Thrombus formation was monitored for 180 seconds in WT mice following laser injury of the cremasteric arteriole in the presence or absence of the platelet aggregation inhibitor eptifibatide (10 μg/g of body weight). (**A**) Representative images at indicated time points of the PS probe annexin V (red, Alexa Fluor 647), platelets (blue, anti-CD42b antibody, DyLight 405), and fibrin (green, anti-fibrin antibody, DyLight 488). Note annexin V positivity on the vessel wall and in the absence of platelet aggregation. Kinetics and magnitude of median integrated RFUs for platelet accumulation (**B**) and PS externalization (**D**) are shown following laser injury. AUC for fluorescence intensity was determined for platelets (**C**) and annexin V (**E**). Lines represent the median AUC for individual thrombi (vehicle *n* = 34, eptifibatide *n* = 35) analyzed by Mann-Whitney *U* test. ****P < 0.0001. A vessel-wall pattern for PS externalization is also observed using alternative PS probes pSIVA (**F**, red pseudocolor) and lactadherin-FITC (**G**, red pseudocolor), shown 180 seconds following laser injury. In both **F** and **G**, platelets are labeled blue, and representative images are shown from 10 individual thrombi. Arrowheads denote extent of vessel-wall injury and x indicates sites of laser ablation. Scale bars: 25 μm.

To directly observe the contribution of vascular PS to thrombus formation in vivo, we used intravital microscopy to monitor PS externalization and platelet and fibrin accumulation in arterioles following laser injury to the vessel wall. The kinetics, localization, and AUC for PS were assessed by injecting fluorescent-conjugated annexin V. Following laser injury, PS externalization was consistently detected along the vessel wall, not in the growing platelet aggregate, and increased gradually, usually plateauing by 180 seconds (Figure 3, A–E, Supplemental Figure 5, and Supplemental Video 1). PS externalization consistently extended proximal and distal to the site of laser ablation and spread to the opposite wall in approximately 20% to 33% of injuries (Supplemental Figure 5). Addition of the integrin α_2b_β_3_ (glycoprotein IIb/IIIa) antagonist eptifibatide prevented platelet accumulation at the site of injury, but had no effect on total PS externalization (Figure 3, B–E, Supplemental Figure 5, and Supplemental Video 2). We employed annexin V at a dose of 0.025 μg/g of body weight, which was significantly lower than annexin V doses shown to inhibit thrombosis in other models (16, 17). Annexin V at this concentration resulted in a mild reduction in fibrin formation, with no effect on platelet accumulation (Supplemental Figure 6). Use of other PS probes such as lactadherin and pSIVA demonstrated an identical vessel wall pattern of PS exposure following laser injury (Figure 3, F and G).

### Mice lacking TMEM16E have reduced fibrin formation following vessel injury.

**Figure 4:**
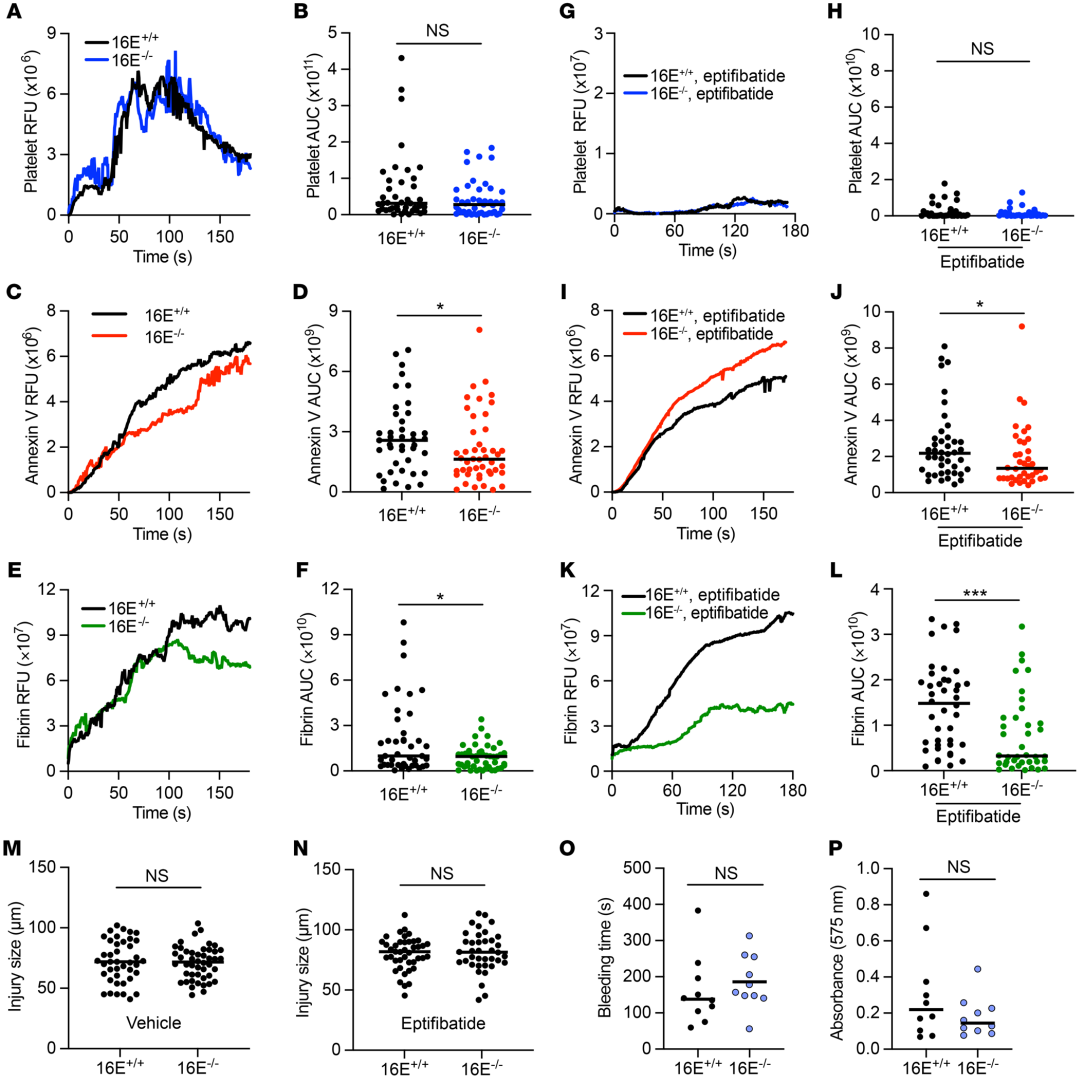
TMEM16E supports vessel-wall fibrin formation. Thrombus formation following laser injury of the cremasteric arteriole was monitored for 180 seconds in TMEM16E^–/–^ (*Ano5^–/–^*) or TMEM16E^+/+^ (*Ano5^+/+^*) littermate controls in the presence of vehicle (**A**–**F** and **M**) or eptifibatide (10 μg/g of body weight) (**G**–**L** and **N**). Platelet and fibrin accumulation were monitored by anti-CD42b and anti-fibrin antibody conjugated to DyLight 405 and 488, respectively. PS externalization was monitored with annexin V conjugated to Alexa Fluor 647. Kinetics and magnitudes of median integrated RFUs for platelet (**A** and **G**), annexin V (**C** and **I**), and fibrin (**E** and **K**) accumulation are shown following laser injury. AUC for fluorescence intensity was determined for platelets (**B** and **H**), annexin V (**D** and **J**), and fibrin (**F** and **L**) for each thrombus. Lines represent the median AUC for individual thrombi (vehicle 16E^+/+^ *n* = 41, 16E^–/–^ *n* = 46; eptifibatide 16E^+/+^ *n* = 42 16E^–/–^ *n* = 38) analyzed by Mann-Whitney *U* test, *P < 0.05; ***P < 0.001. Injury sizes associated with the thrombi analyzed above under vehicle (**M**) and eptifibatide (**N**), analyzed by Student’s *t* test. Time to cessation of bleeding (**O**) and total hemoglobin loss (**P**) were measured following tail transection (16E^+/+^ *n* = 10, 16E^–/–^ *n* = 10), analyzed by Mann-Whitney *U* test.

Since we identified a role for TMEM16E in regulating EC procoagulant activity, we asked whether absence of TMEM16E in mice altered thrombosis. Encoded by *Ano5*, TMEM16E is highly expressed in skeletal muscle, but we have found it is also expressed in ECs (Supplemental Figure 1) (43, 46). TMEM16E^–/–^ mice are overtly healthy but demonstrate defective muscle repair (38). Blood coagulation in TMEM16E^–/–^ mice has not been studied. We used intravital microscopy following laser injury of the cremasteric arteriole to determine whether TMEM16E regulated thrombus formation. TMEM16E^–/–^ mice demonstrated no difference in platelet accumulation compared with TMEM16E^+/+^ littermate controls, but did demonstrate a small but significant decrease in PS externalization, as determined by annexin V fluorescence (median annexin V AUC 2.6 × 10^9^ relative fluorescent units [RFU] for TMEM16^+/+^ versus 1.6 × 10^9^ for TMEM16E^–/–^, *P <* 0.05), and fibrin formation (median AUC 8.3 × 10^9^ RFU for TMEM16^+/+^ versus 7.9 × 10^9^ for TMEM16E^–/–^, *P <* 0.05) (Figure 4, A–F and M). To eliminate the contribution of platelet-mediated thrombosis and to clarify the contribution of platelet versus vessel wall PS, animals were treated with eptifibatide to prevent platelet accumulation. Under eptifibatide treatment, TMEM16E^–/–^ mice still demonstrated a reduction in PS externalization (median annexin V AUC 2.1 × 10^9^ RFU for TMEM16^+/+^ versus 1.4 × 10^9^ for TMEM16E^–/–^, *P <* 0.05) and a larger reduction in fibrin formation (median AUC 1.5 × 10^10^ RFU for TMEM16^+/+^ versus 0.32 × 10^10^ for TMEM16E^–/–^, *P <* 0.001) following laser injury compared with littermate controls (Figure 4, G–L and N). The diminished fibrin deposition in TMEM16E^–/–^ mice could not be attributed to differences in baseline ex vivo coagulation parameters or platelet count (Supplemental Figure 7, A–E). To assess whether TMEM16E was required for hemostasis, we tested 8- to 10-week-old TMEM16E^–/–^ and TMEM16E^+/+^ littermate control mice in a tail-clip–bleeding assay. TMEM16E^–/–^ mice did not demonstrate excessive bleeding following tail transection (Figure 4, O and P).

### TMEM16 inhibitors reduce EC procoagulant activity.

**Figure 5:**
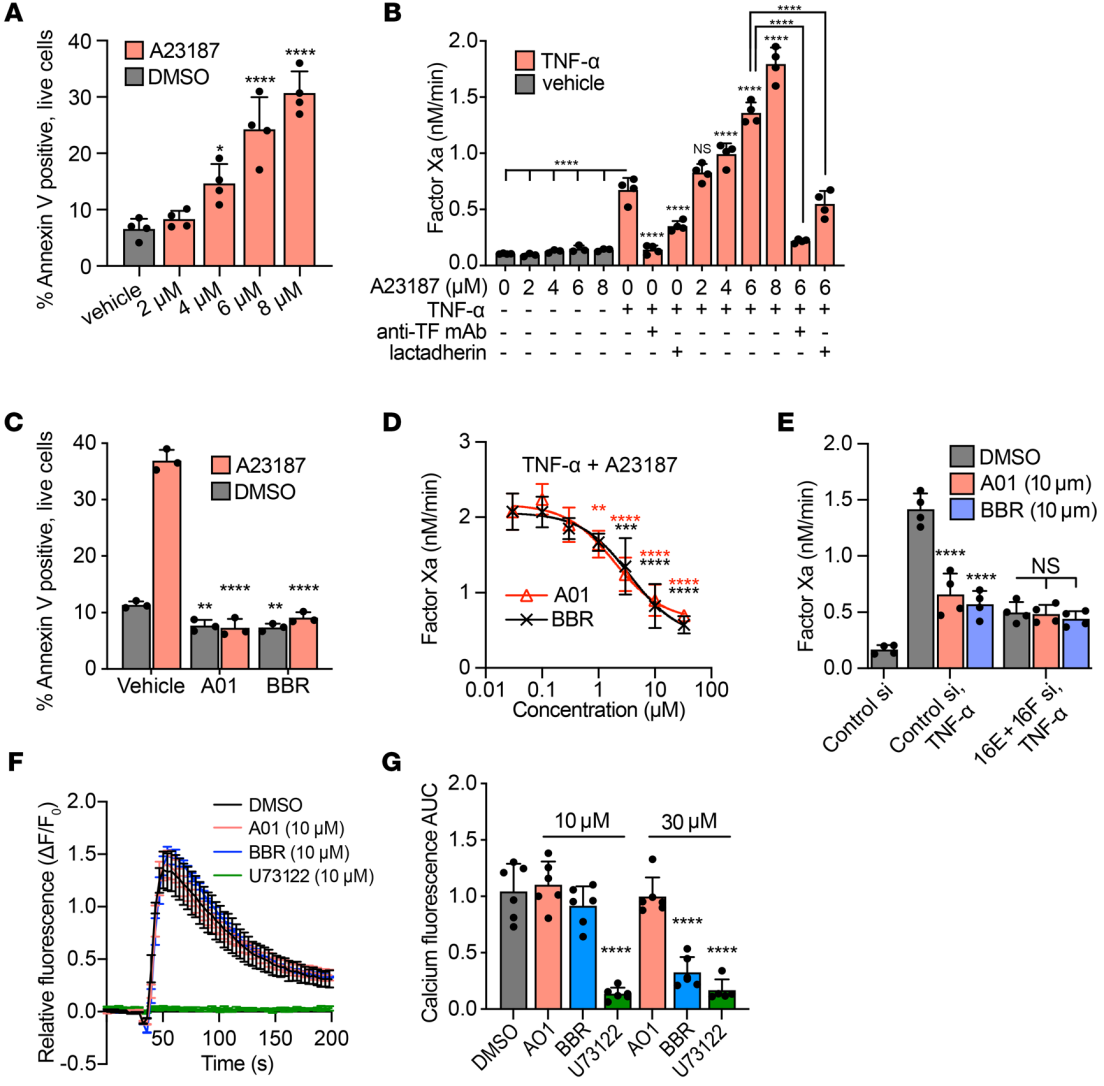
TMEM16 antagonists reduce EC procoagulant activity by inhibiting PS externalization. (**A**) HUVECs were treated with Ca^2+^ ionophore A23187 for 20 minutes, and PS externalization was measured by flow cytometry. Histograms were generated after gating on live (DAPI negative) cells only. (**B**) HUVECs were treated with TNF-α (10 ng/mL) for 3.5 hours and/or A23187 for 20 minutes at indicated concentrations and analyzed for factor VIIa–catalyzed activation of factor X. Anti-TF antibody (10 μg/mL) and lactadherin (100 nM) were used to block TF and PS, respectively. All cells not receiving anti-TF antibody were treated with IgG isotype control. Asterisks denoting statistical significance show comparison with cells treated with TNF-α, no ionophore, unless otherwise specified, with brackets to indicate pairwise comparison. (**C**) HUVECs were treated with A23187 (6 μM) for 20 minutes in the presence of TMEM16 inhibitors CaCCinh-A01 (A01) and BBR (both 10 μM) and analyzed for PS externalization as in **A**. (**D**) TMEM16 antagonists were assayed for their ability to inhibit factor VIIa–catalyzed activation of factor X on HUVECs stimulated with TNF-α (10 ng/mL, 3.5 hours) followed by A23187 (6 μM, 20 minutes). (**E**) HUVECs were transfected with indicated siRNAs, treated with A01 or BBR, stimulated with TNF-α (10 ng/mL, 3.5 hours), and assayed for their ability to support factor Xa generation. (**F** and **G**) HUVECs were treated with A01 or BBR or the phospholipase C inhibitor U73122, and intracellular Ca^2+^ transients were measured with Calbryte 520 AM following stimulation with thrombin (1 U/mL). Time course of Calbryte 520 fluorescence, normalized to background (**F**), and AUC values (**G**), normalized to vehicle-treated cells, are shown. *n* = 3–6 independent experiments. Error bars indicate mean ± SD (**A**–**E** and **G**) or mean ± SEM (**F**). ANOVA with Tukey’s post test. *P < 0.05; **P < 0.01; ***P < 0.001; ****P < 0.0001.

Ca^2+^ ionophore induced PS externalization on ECs in a dose-dependent manner (Figure 5A). PS externalization alone did not promote factor VIIa–catalyzed activation of factor X, presumably due to an absence of TF expression on the endothelial surface (Figure 5B). However, following stimulation with TNF-α, ionophore A23187 promoted a synergistic, dose-dependent increase in factor Xa generation (Figure 5B). This augmentation of procoagulant activity was TF and PS dependent, as it was inhibited with anti-TF antibody or lactadherin (Figure 5B). We tested to determine whether pharmacologic blockade of TMEM16 reduced EC procoagulant activity. CaCCinh-A01 and benzbromarone (BBR) are unrelated small molecules that have broad activity against TMEM16 channels, including TMEM16E and TMEM16F (46–51). Both compounds completely inhibited Ca^2+^ ionophore–stimulated PS externalization in HUVECs at 10 μM (Figure 5C). CaCCinh-A01 and BBR each inhibited ionophore-augmented activation of factor X in a dose-dependent manner with an IC_50_ of approximately 2.0 μM and approximately 3.2 μM, respectively (Figure 5D). To determine whether these compounds inhibited endothelial procoagulant activity independently of TMEM16 proteins, ECs were treated with CaCCinh-A01 or BBR following silencing of TMEM16E or TMEM16F. These compounds did not further inhibit factor Xa generation in the absence of TMEM16E or TMEM16F (Figure 5E). Given the ability of TMEM16 antagonists to affect intracellular Ca^2+^ flux and therefore regulate PS externalization indirectly (51), we tested to determine whether CaCCinh-A01 and BBR reduced procoagulant activity primarily via suppression of intracellular Ca^2+^ elevation. At 10 μM, neither CaCCinh-A01 nor BBR inhibited intracellular Ca^2+^ flux in ECs, in contrast to the phospholipase C inhibitor U73122, which completely abolished intracellular Ca^2+^ elevation in response to thrombin (Figure 5, F and G). BBR did reduce Ca^2+^ transients at higher concentrations; however, CaCCinh-A01 did not (Figure 5G). Overall, these results suggest TMEM16 antagonism with CaCCinh-A01 and BBR reduced procoagulant activity in ECs primarily by inhibiting PS externalization.

### The TMEM16 inhibitor BBR protects against thrombosis.

**Figure 6:**
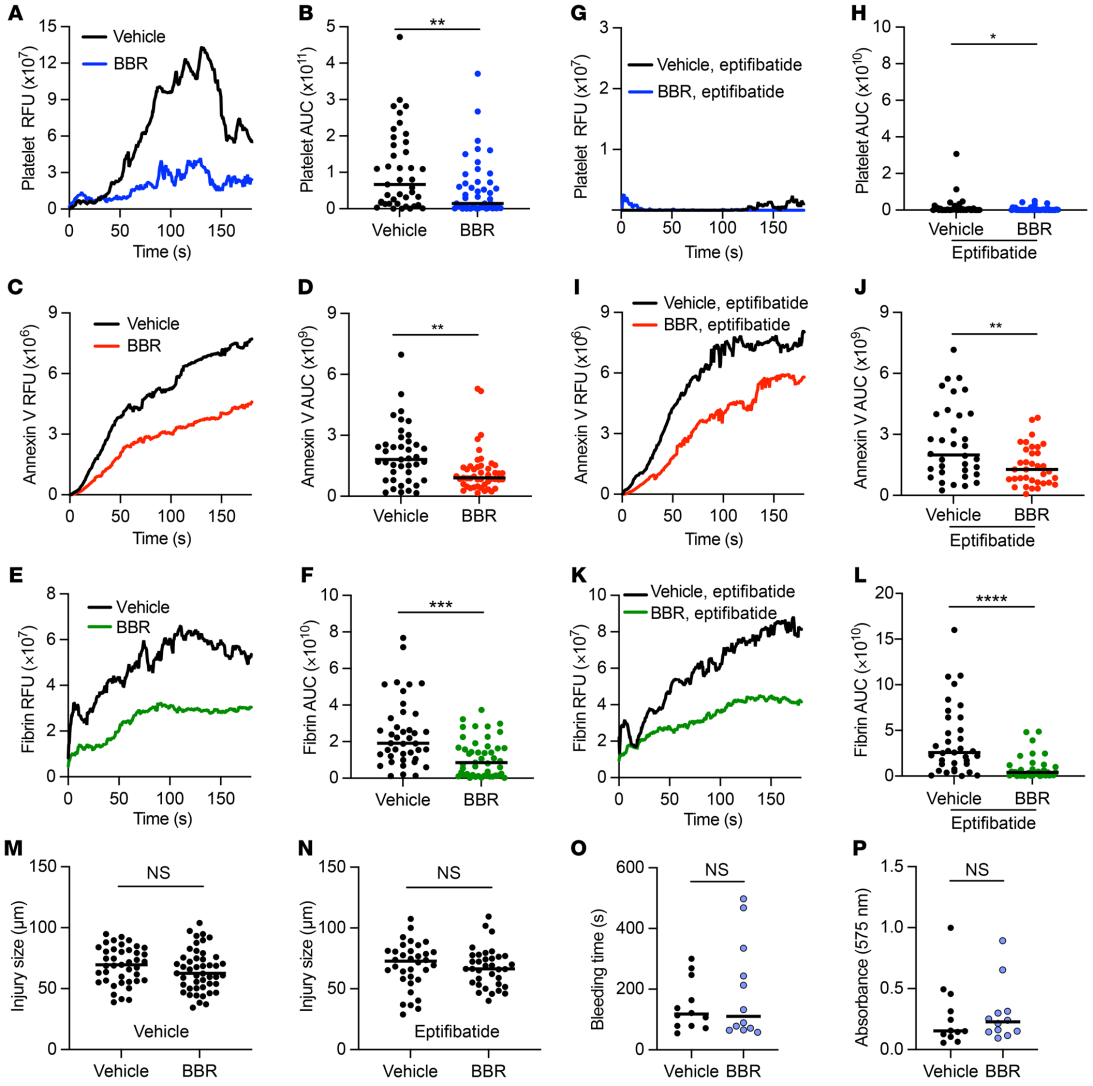
BBR inhibits thrombosis without increasing bleeding following tail transection. Thrombus formation following laser injury of the cremasteric arteriole was monitored for 180 seconds in mice treated with BBR (5 μg/g of body weight) or vehicle (**A**–**F** and **M**) and BBR or vehicle in the presence of eptifibatide (10 μg/g of body weight) (**G**–**L** and **N**). Platelet and fibrin accumulation were monitored by anti-CD42b and anti-fibrin antibody conjugated to DyLight 405 and 488, respectively. PS externalization was monitored with annexin V conjugated to Alexa Fluor 647. Kinetics and magnitude of median integrated RFUs for platelet (**A** and **G**), annexin V (**C** and **I**), and fibrin (**E** and **K**) accumulation are shown following laser injury. AUC for fluorescence intensity was determined for platelets (**B** and **H**), annexin V (**D** and **J**), and fibrin (**F** and **L**) for each thrombus. Lines represent median AUC for individual thrombi (vehicle *n* = 41, BBR *n* = 47; eptifibatide + vehicle *n* = 34 eptifibatide + BBR *n* = 35) analyzed by Mann-Whitney *U* test. *P < 0.05; **P < 0.01; ***P < 0.001; ****P < 0.0001. (**M** and **N**) Injury sizes associated with the thrombi analyzed above, analyzed by Student’s *t* test. Time to cessation of bleeding (**O**) and total hemoglobin loss (**P**) were analyzed following tail transection in mice treated with BBR (vehicle *n* = 12, BBR *n* = 10), analyzed by Mann-Whitney *U* test.

Since there is extensive clinical experience using BBR for the treatment of gout (52), we determined whether BBR demonstrated antithrombotic properties in vivo. WT C57BL/6J mice were treated with intraperitoneal injection of BBR (5 μg/g of body weight) 1 hour prior to evaluating thrombus formation following laser injury of the cremasteric vasculature. BBR reduced platelet accumulation (median AUC 6.7 × 10^10^ RFU for vehicle versus 1.5 × 10^10^ for BBR, *P <* 0.01), PS externalization (median annexin V AUC 1.8 × 10^9^ RFU for vehicle versus 0.91 × 10^9^ for BBR, *P <* 0.01), and fibrin formation (median AUC 1.9 × 10^10^ RFU for vehicle versus 0.85 × 10^10^ for BBR, *P <* 0.001) following vessel injury (Figure 6, A–F and M). When mice were treated with eptifibatide to prevent platelet aggregation, BBR still reduced PS externalization (median annexin V AUC 2.0 × 10^9^ RFU for vehicle versus 1.3 × 10^9^ for BBR, *P <* 0.01) and fibrin formation (median AUC 2.6 × 10^10^ RFU for vehicle versus 0.4 × 10^10^ for BBR, *P <* 0.0001) following laser ablation (Figure 6, G–L and N). BBR treatment did not affect coagulation parameters or result in increased bleeding following tail transection (Figure 6, O and P, and Supplemental Figure 7, F and G).

## Discussion

Biochemical and in vitro studies have established that membranes containing anionic phospholipid, most notably PS, are essential for enabling blood coagulation at physiologic reaction kinetics (2). How PS externalization operates in vivo to support hemostasis and thrombosis is not well understood because exposure of PS is not captured by routine clinical assays and investigation of procoagulant PS has largely focused on platelets or other blood cell components. Our data suggest that the vessel wall is a major source of PS externalization to support thrombus formation. Previous mechanistic studies on PS externalization in blood coagulation have largely been limited to a single PLS, TMEM16F. Here, we identify another PLS, TMEM16E, as a positive regulator of PS externalization and procoagulant activity in ECs that may participate in thrombosis. TMEM16 inhibitors decrease EC procoagulant activity and protect against thrombosis without increased bleeding following tail transection.

As cellular mediators of hemostasis and thrombosis, some activated platelets form membrane blebs containing externalized PSs that support thrombin generation (53). Other blood-contacting cells, including monocytes (54), erythrocytes (55, 56), and ECs (27, 57, 58), can also support coagulation in a PS-dependent manner. The laser-ablation model of vascular injury coupled with intravital microscopy has the ability to discern vessel wall–mediated versus platelet-mediated contributions to thrombosis (28, 59–61). Employing 3 different PS probes, we observed that, following vessel injury, accessible PS was detected on the vessel wall, not platelets, despite the formation of large platelet aggregates (Figure 3, Supplemental Figure 5, and Supplemental Video 1). While unexpected, our results are consistent with prior studies demonstrating that coagulation enzyme complex assembly and fibrin generation occur along the vessel wall and can be independent of platelet accumulation (60–62). We readily detected both PS externalization and fibrin formation on the vessel wall despite inhibition of platelet accumulation with eptifibatide (Figures 3, Figure 4, Figure 6, Supplemental Figure 5, and Supplemental Video 2). Our data therefore expand current models of thrombosis and support a framework where the activated endothelium promotes clotting via PS externalization.

The degree to which laser ablation results in endothelial disruption versus activation is not known, and these effects are likely to vary among injuries. Similarly to certain pharmacologic stimuli, laser pulses induce a rapid Ca^2+^ elevation in ECs that propagates to cellular neighbors (63) and likely explains the proximal, distal, and sometimes circumferential extension beyond the site of laser ablation (Figure 3 and Supplemental Figure 5). However, platelet accumulation and fibrin formation also require exposure of adhesion proteins and TF, respectively. Therefore, PS externalization is necessary but not sufficient for thrombus formation in this model. PS externalization crosses over to the vessel wall where laser ablation is performed in approximately 20% to 33% of injuries, often in vessels of smaller diameter, and results in a larger measured sum fluorescent intensity independent of injury size (Supplemental Figure 8C). This observation is in contrast to fibrin or platelet accumulation, which correlate well with injury size (Supplemental Figure 8, A and B) (64). The lack of correlation between annexin V sum intensity and laser-induced injury size is due to limitations of quantifying annexin V in a single *z* plane and related to characteristics of the cremaster prep (e.g., tissue thickness and vessel depth) and vessel diameter, rather than a biologic variable (e.g., TMEM16E genotype, eptifibatide, and/or BBR treatment). Correlation of annexin V fluorescence with injury size improved when injuries with excessive crossover of annexin V binding were removed from analysis (Supplemental Figure 8D). Despite these caveats, annexin V qualitative staining was very consistent, and the progression of PS externalization away from the site of injury occurred universally following laser ablation of all intensities.

Inflammatory stimuli such as lipopolysaccharide (28) or TNF-α (this study) led to concurrent expression of TF and externalization of PS, which together support procoagulant activity. This procoagulant activity was augmented by further externalization of PS (Figure 5B). PS externalization in ECs, similar to that in other cells, including platelets, results from TMEM16 activation via sustained elevations in intracellular Ca^2+^ (11). Thus, TMEM16E and TMEM16F couple Ca^2+^ signaling to procoagulant activity in ECs. Some data suggest that TMEM16E and TMEM16F influence Ca^2+^ flux itself (45), whereas our data in ECs are similar to those in studies of platelets and lymphocytes from patients with Scott syndrome, where TMEM16F deficiency abolishes PS externalization without inhibiting intracellular Ca^2+^ elevation (65, 66). While apoptosis of ECs results in PS externalization, and therefore procoagulant activity (57), the TNF-α stimulation used in our studies did not induce EC death (Figure 2 and Supplemental Figure 4). Viable cells can expose PS (67), and this exposure is reversible (9, 27), as flippase activity of P4-ATPases restores lipid asymmetry (42). Our targeted screen suggested the flippase ATP11C may be a negative regulator of EC procoagulant activity, although the result did not meet the threshold for statistical significance (Figure 1A). PS exposure on the endothelium may be a mechanism by which both apoptotic and nonapoptotic inflammatory stimuli modulate coagulation reactions, and it is possible that other mechanisms of procoagulant PS externalization in ECs are relevant in different pathophysiologic states.

The TMEM16 family has 10 members, all of which act as Ca^2+^-activated ion channels, PLSs, or in certain cases, perhaps both (36). The physiologic function of many family members remains unknown. Structural and biochemical studies establish a distinct “scrambling” domain of TMEM16F and its closest paralog, TMEM16E, that allows for transmembrane phospholipid exchange (37, 43, 44, 68, 69). TMEM16E regulates muscle regeneration, myoblast fusion, and myocyte membrane repair (37, 38, 70). Curiously, one function of TMEM16E, membrane repair following injury, appears to be independent of scramblase domain function (68). Mutations in TMEM16E cause limb girdle muscular dystrophy type 2L (LGMD2L) (71), an autosomal recessive, late-onset muscular dystrophy, or gnathodiaphyseal dysplasia (GDD) (72), an extremely rare autosomal dominant pediatric bone disease. Neither of these conditions has been associated with abnormal hemostasis or thrombosis, but this possibility has not been investigated.

A series of investigations has demonstrated that TMEM16E readily scrambles phospholipid, but the localization of TMEM16E within cells and importance of TMEM16E lipid scrambling per se is unclear (37, 43). Some studies suggest TMEM16E is predominantly expressed in intracellular compartments, such as the ER (43, 73, 74), whereas others suggest it may also be located on the plasma membrane (37, 75). Of relevance to our own thrombosis model, one study in myocytes found that laser injury induced redistribution of TMEM16E to the plasma membrane within seconds (68). The mechanism by which TMEM16E regulates PS externalization on the plasma membrane is therefore incompletely understood (37). TMEM16E-null animals do not compensate by increasing expression of TMEM16F (68, 76). Dual silencing of both TMEM16E and TMEM16F does not result in additive reduction in procoagulant activity, likely because silencing of either TMEM16E or TMEM16F nearly completely abolishes PS externalization (Figure 1 and Figure 2). The requirement of both TMEM16E and TMEM16F for PS externalization in ECs is unclear, but a similar phenomenon has been found in skeletal muscle cells, which express high levels of TMEM16E (37). This observation raises the possibility that TMEM16E and TMEM16F are epistatic to one another, perhaps regulating PS externalization through direct interaction or through a pathway involving both proteins, which will be the focus of future investigation.

Prior studies have evaluated the role of TMEM16F in thrombosis, but have focused on platelets. Global TMEM16F-null mice demonstrate delayed occlusion in a ferric chloride carotid injury model of thrombosis and a mild bleeding diathesis (20). Animals with a platelet-specific conditional TMEM16F deletion exhibit reduced platelet aggregates in a reactive oxygen species–based model of mesenteric venule thrombosis (22) and are protected from ferric chloride carotid thrombosis but not from thromboinflammatory ischemic stroke (23). In contrast, platelet-specific deletion of phosphatidylinositol transfer protein α (PITPα) abolishes PS externalization in platelets due to a lack of IP_3_-induced Ca^2+^ elevation, but has no effect on thrombosis in a ferric chloride model (24). These studies support the possibility that sources beyond the platelet, such as the endothelium and regulators other than TMEM16F, control PS externalization in hemostasis and thrombosis.

We find that 2 unrelated compounds, CaCCinh-A01 and BBR, reduce endothelial PS externalization and procoagulant activity. While there is extensive evidence these drugs exert physiologic effects via TMEM16 antagonism, we cannot rule out an additional effect on coagulation via mechanisms independent of TMEM16E or TMEM16F (46–51). One such agent, the uricosuric agent BBR, has been used for decades to treat gout worldwide (77, 78). BBR is generally considered safe but was not approved by the FDA due to serious but exceedingly rare hepatotoxicity events estimated at 1 in 17,000 (52). Nevertheless, TMEM16 inhibition remains a compelling antithrombotic strategy to explore, perhaps as prophylaxis to mitigate PS externalization during inflammation or in conjunction with traditional anticoagulants in cases of refractory thrombosis.

In summary, this investigation establishes TMEM16 proteins as drivers of EC procoagulant activity. We specifically identify 2 PLSs, TMEM16F, known to contribute to procoagulant activity in platelets, and TMEM16E, which has not been previously implicated in coagulation. We provide evidence that the vessel wall contributes procoagulant phospholipids to support thrombosis. Moreover, fibrin generation, the terminal product in coagulation activation, is impaired in TMEM16E-deficient mice following vascular injury. We demonstrate the potential of TMEM16 antagonism to dampen prothrombotic endothelium and inhibit thrombosis without increasing bleeding complications. These observations suggest new areas of investigation into the role of endothelial membrane phospholipid dynamics in coagulation and the potential of TMEM16 inhibition for treatment of pathologic blood clotting.

## Methods

### Cell culture and siRNA transfection.

HUVECs (pooled donor, Lonza) were grown and maintained in EC growth basal media (EBM-2, Lonza) containing 2% FBS and contents of the EGM-2 SingleQuots Growth Factor Supplement Kit. Cells from passages 3 to 5 were used for experiments. Cells were reverse transfected with siRNA at a final concentration of 20 nM (96-well plate) or 40 nM (384-well plate) using Lipofectamine RNAiMax (Thermo Fisher Scientific) according to the manufacturer’s protocol. The following siRNA sequences were used: (Dharmacon siGENOME or ON-TARGET) TMEM16E (*ANO5*), CUACGUAGCUUUCUUUAAA and GCACACUCCUAUAAGCUAU; TMEM16F (*ANO6*), GAUCAUCGCUUCAGUUAUU, CAACUCAGCUGACAAUAAU; TF (*F3*), CAUUGGAGCUGUGGUAUUU; nontargeting SIGENOME siRNA pool, UAAGGCUAUGAAGAGAUAC, AUGUAUUGGCCUGUAUUAG, AUGAACGUGAAUUGCUCAA, and UGGUUUACAUGUCGACUAA. Dharmacon siGENOME SMARTpool siRNA, a mixture of 4 siRNAs, was used for *ATP8A2*, *ATP11A*, *ATP11C*, *CDC50A*, *STIM1*, *TFPI*, *TMEM16C*, *TMEM16D*, *TMEM16E*, *TMEM16F*, *TMEM16G*, *TMEM16K*, *XKR4*, *XKR8*, and *XKR9*.

### Factor X activation and thrombin-generation assays.

Primary HUVECs or EA.hy926-TF^hi^ cells were plated in 384-well or gelatin-coated 96-well plates. For experiments using siRNA transfection, 1,000 (384-well plate) or 5,000 (96-well plate) cells were transfected, plated, and allowed to grow for 72 hours. For all other experiments, 20,000 cells were plated (96-well plate) 1 day before the experiment. Where indicated, cells were stimulated with TNF-α (10 ng/mL, R&D Systems) for 3.5 hours and/or Ca^2+^ ionophore A23187 (MilliporeSigma) for 20 minutes at 37°C, washed twice with HBS-BSA (20 mM HEPES, pH 7.4, 150 mM NaCl, 5 mM KCl, 5 mM CaCl_2_, 1 mg/mL fatty acid–free BSA, MilliporeSigma), and equilibrated to room temperature. For factor X activation (factor Xa generation) experiments, cells were incubated with HBS-BSA containing factor X (125 nM, Haematologic Technologies), factor VIIa (0.6 nM, Haematologic Technologies), and factor Xa chromogenic substrate Biophen-CS11 (22) (150 μM, Aniara Diagnostica). Absorbance at 405 nm was measured every minute for 3 hours on an xMark Spectrophotometer (Bio-Rad). Maximal reaction velocity was converted to factor Xa nM/min based on standard curve analysis of purified factor Xa (Haematologic Technologies) serial dilutions with the same reaction conditions. In the focused siRNA screen of PLSs (Figure 1A), cells were transfected with the corresponding Dharmacon siGENOME gene-specific siRNA pool of 4 siRNAs in an 384-well format and assayed as described above, except that a single absorbance at 405 nm was determined at 45 minutes on an EnVision plate reader (PerkinElmer) and presented as a percentage of untargeted siRNA control. For experiments using CaCCinh-A01 or BBR (Cayman Chemical), drug (0.03-30 μM) or DMSO vehicle control was added at the time of TNF-α stimulation. When TF inhibitory antibody (10 μg/mL, clone 4509, BioMedica Diagnostics), mouse IgG1 isotype control (10 μg/mL, Thermo Fisher Scientific), or bovine lactadherin (100 nM, Haematologic Technologies) was evaluated, cells were washed in HBS-BSA and incubated in the presence of indicated proteins in HBS-BSA for 10 minutes. The reaction was then triggered by addition of factors X and VIIa and factor Xa substrate at final concentrations indicated above. For thrombin-generation experiments, cells were washed twice with HBS-BSA and incubated in 80 μL HBS plus 20 μL pooled human plasma (George King Bio-Medical) to supply coagulation factors, H-Gly-Pro-Arg-Pro-OH fibrin polymerization inhibitor (GPRP, 5 mM, Cayman Chemical), and fluorogenic thrombin substrate Boc-L-FPR-ANSNH-C_2_H_5_ (50 μM, SN-20, Haematologic Technologies). In certain samples, bovine lactadherin (100 nM, Haematologic Technologies) was added to the reaction mixture. The reaction was initiated by addition of 0.8 to 1.1 mM CaCl_2_ and read immediately using a Synergy HTX plate reader (BioTek). Fluorescence (excitation 352 nm/emission 470 nm) was measured every minute for 1 hour. The first derivative of thrombin-generation curves was compared with a standard curve of thrombin to determine thrombin generated in U/mL. All individual experiments were performed in technical triplicate, and the mean of these technical replicates was used as the value for each independent experiment.

### Immunofluorescence microscopy.

Following siRNA transfection, HUVECs were plated directly in gelatin-coated glass chamber slides for 72 hours. Cells were washed with 10 mM HEPES buffer (pH 7.4) containing 140 mM NaCl, 2.5 mM CaCl_2_ (annexin V binding buffer), and stained with annexin V conjugated to Alexa Fluor 488 (Thermo Fisher Scientific) at a 1:50 dilution and Zombie Red viability dye (BioLegend) at a 1:1,000 dilution in the dark for 15 minutes at room temperature in annexin V–binding buffer. Cells were washed and fixed in annexin V–binding buffer containing 4% paraformaldehyde for 7 minutes. Cells were washed 3 times and mounted with DAPI (ProLong Gold Antifade Mountant, Thermo Fisher Scientific). For TF immunofluorescence staining, cells were washed 3 times with PBS and fixed in PBS containing 4% paraformaldehyde for 7 minutes. To avoid permeabilizing cells, no detergents were added in the blocking or washing steps. Cells were blocked in PBS containing 10% goat serum (Jackson ImmunoResearch) and 1% BSA for 1 hour and then stained in blocking solution containing anti-TF antibody (5 μg/mL, clone 4509, BioMedica Diagnostics) overnight at 4°C. Cells were washed 3 times in PBS and stained with anti-mouse secondary antibody conjugated to Alexa Fluor 488 (Jackson ImmunoResearch) at a dilution of 1:500 for 1 hour at room temperature. Cells were washed 3 times in PBS and mounted in DAPI. Images were obtained using a Zeiss LSM 880 upright laser scanning confocal microscope in 3 × 3 tile-scan mode with a Plan-Apochromat ×20/0.8 M27 objective. Fluorescent images were analyzed using ImageJ software (NIH). For PS externalization, the threshold for annexin V staining was equalized for all images and total fluorescent area was obtained, minus Zombie Red fluorescent area, and normalized to the number of nuclei. For TF immunofluorescence, mean fluorescence intensity was quantified per tissue area after measuring and subtracting background signal from each image.

### Flow cytometry.

HUVECs were stimulated with indicated concentrations of Ca^2+^ ionophore A23187 (MilliporeSigma) for 15 minutes at 37°C and washed with PBS. For experiments using CaCCinh-A01 or BBR (Cayman Chemical), drug (10 μM) or DMSO vehicle control was added 90 minutes prior to A23187 stimulation. Cells were dissociated using Accutase (STEMCELL Technologies) and washed by repelleting twice in cold PBS. Cells (~1 × 10^6^ per mL) were resuspended in 10 mM HEPES buffer (pH 7.4) containing 140 mM NaCl, 2.5 mM CaCl_2_, and 1 mg/mL BSA, and PS externalization was determined by staining for 15 minutes in the dark with annexin V conjugated to Alexa Fluor 488 (Thermo Fisher Scientific) at a 1:20 dilution plus DAPI solution (0.1 μg/mL, BD Biosciences) as a viability stain. Cells were gated using forward and side scatter, and 10,000 events were collected using a CytoFLEX LX (Beckman Coulter) running CytExpert software, version 2.5. Data were analyzed using FloJo software, version 10.8.1 (BD Biosciences), and annexin V–positive (PS externalized) cells were determined in populations of viable (DAPI-negative) cells only.

### Intracellular Ca^2+^ measurements.

Confluent HUVECs in a 96-well plate were washed with HBSS (140 mM NaCl, 5 mM KCl, 1 mM CaCl_2_, 4 mM MgSO_4_, 5 mM MgCl_2_, 3 mM Na_2_HPO_4_, 4 mM KH_2_PO_4_, 6 mM d-glucose, 4 mM NaHCO_3_) and loaded with 4 μM Calbryte 520 AM (AAT Bioquest) in HBSS for 30 minutes. Calbryte 520 does not bind Ca^2+^ until it is esterified intracellularly and therefore can be loaded in buffers containing divalent cations. After incubation, samples were washed with HBSS and replaced with 100 μL of HBSS. For experiments involving drug treatment of HUVECs, the compounds were added during Calbryte 520 loading for 30 minutes. U73122 was obtained from Tocris. All Ca^2+^ flux assays were performed using the Molecular Devices FlexStation III dual-monochromator plate reader with automated pipetting at the Harvard ICCB-Longwood Screening Facility. Fluorescence was measured every 1.2 seconds on the FlexStation III at Ex/Em = 490/525 nm with a cutoff of 515 nm. After an initial baseline fluorescence reading of 30 seconds, cells were treated with human α-thrombin (Haematologic Technologies) in stimulant volume of 25 μL for a final concentration of 1 U/mL. The 30-second baseline fluorescent readings were averaged (*F_0_*), and the relative change in fluorescence was calculated according to the equation (*F* – *F_0_*)/*F_0_*. The fluorescent readings from technical triplicates of the same condition were averaged and the AUC was calculated.

### Immunoblotting.

HUVEC cultures were lysed using RIPA buffer (Boston Bioproducts) supplemented with cOmplete Protease Inhibitor Cocktail (Roche) and PhosStop Phosphatase Inhibitor Cocktail (Roche), 1 mM Na_3_VO_4_ (New England Biolabs), and 1 mM NaF. Proteins were resolved via SDS-PAGE 4%–12% gradient gels (Thermo Fisher Scientific) under reducing conditions using NuPAGE SDS sample buffer and sample reducing agent (Thermo Fisher Scientific), transferred to a nitrocellulose membrane, and blocked with SuperBlock buffer (Thermo Fisher Scientific). Protein detection was performed with the following primary antibodies: TMEM16E/Ano5 (clone N421A/85, UC Davis/NIH NeuroMab), V5-Tag (80076, Cell Signaling Technology), TMEM16F (MilliporeSigma), TFPI (AF2974, R&D Systems), β-actin, and GAPDH (12620 and 2118, respectively, Cell Signaling Technologies). Appropriate species-specific HRP-conjugated secondary antibodies were also used (Cell Signaling Technologies). Immunoblots were developed with Supersignal West Dura Chemiluminescent Substrate (Thermo Fisher Scientific) and visualized with a GeneGnome XRQ (Syngene) or a ChemiDoc (Bio-Rad) and analyzed using ImageJ software (NIH).

### Intravital microscopy and laser-induced vessel wall–injury model.

Thrombus formation was visualized via intravital microscopy following laser-induced injury to the cremasteric arteriole in male mice as previously described (28, 79). Platelets were detected using anti-CD42b antibody conjugated to DyLight 405 (0.1 mg/g of body weight; clone Xia.G5, Emfret Analytics), fibrin was detected with anti-fibrin antibody (0.5 mg/g of body weight; clone 59D8) conjugated to DyLight 488 (Thermo Fisher Scientific), and PS externalization was detected using annexin V (0.025 μg/g of body weight) conjugated to Alexa Fluor 647 (Thermo Fisher Scientific) infused via internal jugular vein catheter. Annexin V was redosed every 10 to 15 minutes due to its rapid metabolism. For indicated experiments, lactadherin conjugated to FITC (Haematologic Technologies) or pSIVA (Novus Biologicals) was injected intravenously at a dose of 1 μL/g of body weight. For experiments with eptifibatide, mice were injected intravenously with eptifibatide (10 μg/g of body weight, Cayman Chemical) every 10 to 15 minutes. The cremasteric microcirculation was surgically exposed, and injury to the cremasteric arteriole was stimulated with a MicroPoint Laser System (Photonics Instruments). Imaging was performed on a M205 FCA microscope (Leica) with LED-based fluorescence light engine (SpectraX, Lumencor). Data were digitally captured via an Orca Flash 4.0v2 CMOS camera in the 400/420 nm, 488/520 nm, and 647/670 nm fluorescence channels at a rate of 2 frames per second beginning before and extending for 180 seconds after laser injury. Images were analyzed using Slidebook version 6.0 (Intelligent Imaging Innovations). Injury size was determined using calipers 1 frame after laser injury (64). Due to crossover of annexin V binding to the vessel wall opposite of the site of ablation in some injuries and resultant poor correlation of annexin V fluorescence with injury size, we only analyzed injuries where annexin V binding had minimal or no crossover. For each thrombus generated, a rectangular background mask was defined that included a portion of the cremaster prep upstream of the injury that was negative for platelet, annexin, or fibrin staining. The maximum fluorescence intensity of the pixels contained in this mask was extracted for all frames (before and after injury) for each thrombus. The mean value calculated from the maximal intensity values in the mask for each frame was determined and used as the background value. Finally, for each frame, the integrated fluorescence intensity was calculated per the following equation: integrated fluorescence intensity = sum intensity of signal – (mean of the maximal background intensity × area of the signal). This calculation was performed for all frames in each thrombus and plotted versus time to provide the kinetics of thrombus formation. AUC was calculated for individual thrombi to evaluate statistical significance (28, 79). For multiple fluorescence channels, calculations of background were made independently for each channel. At least *n* = 30 injuries across at least 3 mice were used to determine the median value of the integrated fluorescence intensity to account for the variability of thrombus formation at any given set of experimental conditions. The operator was blinded to genotype during injury and image analysis and to drug dosing during image analysis in all experiments.

### Tail transection bleeding assay.

The tail was immersed in saline prewarmed to 37°C for 2 minutes. The tail was then transected 5 mm from the tip and immediately immersed back into 15 mL of warmed saline. The total bleeding time (including rebleeding time) was recorded for 10 minutes. Red blood cells were pelleted by centrifugation at 300*g* for 6 minutes, and the pellet was lysed in 3 mL of red blood cell lysis buffer. The amount of hemoglobin was spectrophotometrically determined by measuring light absorbance at 575 nm.

### Animals.

TMEM16E (Ano5)-null mice on a C57BL/6J background were described previously (38). WT C57BL/6J mice were obtained from Jackson Laboratory. Experiments were performed on mice 8 to 12 weeks of age. For experiments with BBR (Cayman Chemical), the compound was dissolved to a concentration of 40 mg/mL in DMSO and diluted to a working solution in corn oil to a final concentration of 1 mg/mL. BBR or DSMO vehicle control in corn oil was injected intraperitoneally at a concentration of 5 μg/g of body weight 1 hour prior to experiments. Mice were anesthetized with intraperitoneal injection of ketamine (125 μg/g of body weight) and xylazine (12.5 μg/g of body weight) and secured on a heating pad via taping of the paw tips. For intravital microscopy experiments, additional intravenous anesthesia with pentobarbital (5 μg/g of body weight) was administered via internal jugular catheter.

### Statistics.

Tests of normality were performed using the Anderson-Darling and D’Agonstino-Pearson methods. Statistical significance for binary comparisons of continuous variables was assessed by unpaired, 2-tailed Student’s *t* test unless the data did not demonstrate normality, as for intravital imaging data, in which case, differences between groups were analyzed by Mann-Whitney *U* test as previously described (28, 62, 79). For comparison of more than 2 groups, 1-way ANOVA was performed with application of Tukey’s post-test method to adjust for multiple comparisons. Assumptions on the statistical power for rodent experiments were based on our extensive experience with the performance characteristics of the intravital cremasteric laser-injury model and the tail-clip–bleeding assay. All statistical analysis, AUC, and curve fitting was performed using GraphPad Prism (version 9.0; GraphPad Software). *P* values of less than 0.05 were considered significant.

### Study approval.

The Beth Israel Deaconess Medical Center Institutional Animal Care and Use Committee approved all animal care and experimental procedures.

## Author contributions

AAS, HCH, SMP, RF, and SS conceived the study. AAS designed, conducted, supervised, and analyzed experiments. AAS, SS, PFA, SMC, KDS, and EED conducted and analyzed EC experiments. IA and PFA performed mouse hematologic experiments. AAS, PFA, and MPK analyzed intravital microscopy data. The manuscript was written by AAS and SS with input from all of the authors.

## Supplementary Material

Supplemental data

Supplemental video 1

Supplemental video 2

## Version 1 03/23/2023

In-Press Preview

## Version 2 06/01/2023

Electronic publication
