# Supplementary material for: TMEM16E regulates endothelial cell procoagulant activity and thrombosis
Source: J Clin Invest. 2023 Jun 1;133(11):e163808. doi: 10.1172/JCI163808 (PMC10231993; doi:10.1172/JCI163808)

Supplemental Methods

Reagents and cell lines

LPS from *E. coli* serotype O111:B4 was purchased from Sigma. LPS-binding protein and sCD14 were purchased from R&D Systems. Generation of Ea.hy926 cells stably expressing tissue factor was described previously (1). Ea.hy926 cells were cultured in DMEM containing 10% FBS (Gibco) and 5 µg/mL blasticidin (InvivoGen). Human coronary artery endothelial cells (CAEC, single donor, Lonza) and human lung microvascular endothelial cells (HMVEC-L, single donor, Lonza) were grown and maintained in endothelial cell growth basal media (EBM-2, Lonza), containing 2 or 10% fetal bovine serum (FBS) and contents of the EGM-2 or EGM-2 MV SingleQuots growth factor supplement kits, respectively. Cells from passages 3-5 were used for experiments.

Blood and coagulation assays

Hematologic analysis of whole blood counts was performed using a Hemavet 850FS (Drew Scientific) for white blood cell counts, hemoglobin measurement, and platelet counts. The prothrombin time was measured using the Neoplastine CI Plus (Diagnostica Stago, Inc.) and the activated partial thromboplastin time was measured using STA-PTT (Diagnostica Stago, Inc.). Coagulation assays were read on a Start benchtop analyzer (Diagnostica Stago, Inc.) according to manufacturer's instructions.

Quantitative PCR

Gene expression was determined using a 2-step Cell-to-CT kit (ThermoFisher Scientific) with the Hs1381106_m1 (*ANO5*), Hs03805835_m1 (*ANO6*), Hs00372436_s1 and Hs00194899_m1 (*ACTB*) gene expression probes (Taqman, ThermoFisher Scientific). Quantitative reverse transcriptase polymerase chain reaction (qRT-PCR) was performed in technical duplicate for

each biologic sample using a QuantStudio 6 Flex real-time PCR system. Gene expression was compared to *ACTB* expression using the $\Delta\Delta$Ct method.

Lentiviral gene transfer

Full length TMEM16E (*ANO5*) coding sequence (Harvard Plasmid Core, clone Hs CD00345649) was subcloned into the lentiviral vector pLX304 (Addgene plasmid #25890, gift from David Root). Lenti-X 293T cells (Takara) were cotransfected with ANO5-pLX304 lentiviral transfer vector and packaging vectors (pMD2.G and psPAX2, Addgene plasmids #12259 and #12260, respectively, gift from Didier Trono) using Lipofectamine 3000 (ThermoFisher Scientific). Beginning after 48 hours, the viral supernatant was collected twice every 24 hours and replaced with fresh DMEM supplemented with 10% fetal bovine serum. Primary HUVECs (passage 1-2) were cultured in the presence of viral supernatant for 6 hours and then replaced with complete endothelial cell growth media (see Endothelial cell culture and siRNA transfection) for 48 hours before selecting for transduced cells with addition of blasticidin (10 µg/mL) to the cell culture media.

Cell Viability Assay

To assess cell viability of cells transfected with indicated siRNAs, an XTT Cell Proliferation Assay (ATCC) was performed according to manufacturer protocol. Absorbance at 630 nm and 450 nm were measured on an xMark Spectrophotometer (Bio-Rad).

Confocal intravital microscopy for Z-stack images

Wild-type C57BL/6J male mice were anesthetized and prepped as described in “Intravital microscopy and laser-induced vessel wall injury model” and the cremaster arteriole was injured using the Ablate! (3i) laser ablation system. Z-stack images were obtained on an CSU-W1 spinning disk confocal microscope with SoRa super resolution optical unit (Yokagawa) using a

6-line laser illumination system (3i), 63X high numerical aperture water corrected lens (Zeiss) and Orca-Fusion BT sCMOS digital video camera (Hamamatsu). Z-stack images were rendered into 3-dimensional images using using Slidebook version 6.0 (Intelligent Imaging Innovations).

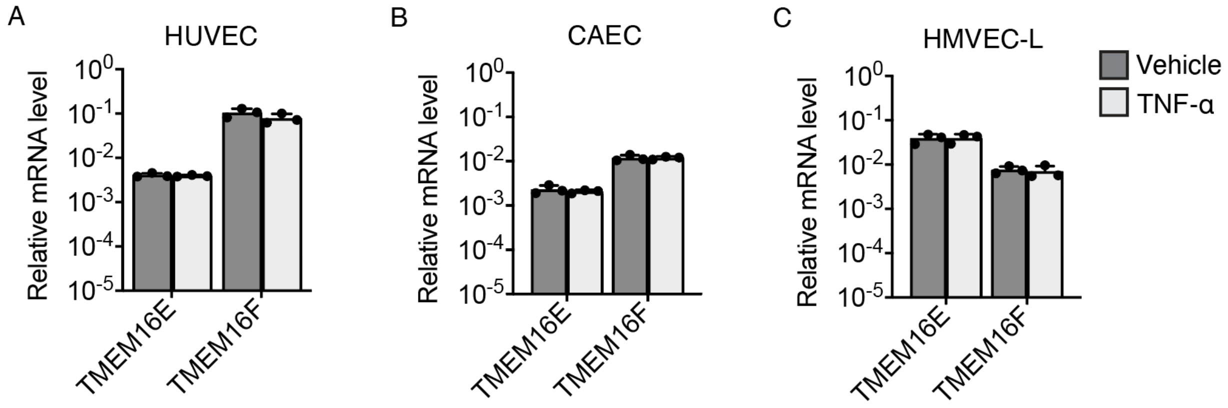

Supplemental Figure 1. Expression of TMEM16E and TMEM16F in primary endothelial cells. mRNA from HUVECs (**A**), coronary artery endothelial cells (CAEC, **B**) and lung microvascular endothelial cells (HMVEC-L, **C**) following stimulation with TNF-α (10 ng/mL) was analyzed by qPCR. Relative mRNA level is compared to that of β-actin. n = 3 independent experiments, Error bars indicate mean ± SD.

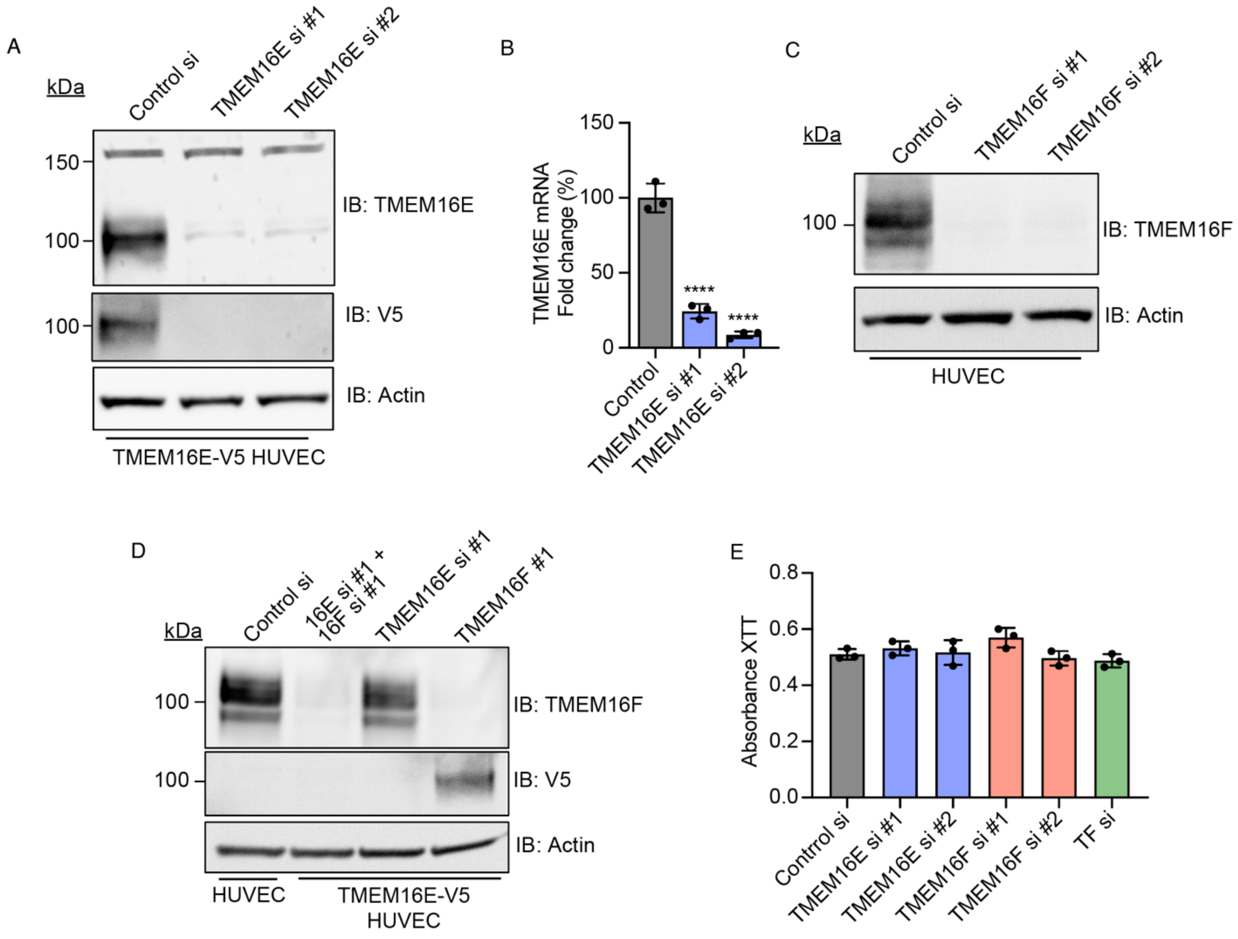

Supplemental Figure 2. Validation of siRNA targeting TMEM16E and TMEM16F. A. HUVECs stably expressing TMEM16E containing a C-terminal V5 tag were transfected with indicated siRNA targeting TMEM16E or untargeted control siRNA for 72 h. TMEM16E protein was determined by SDS-PAGE and immunoblotting with antibodies against TMEM16E, V5, and actin (loading control). TMEM16E is detected as a band running just above 100 kd. **B.** Primary HUVECs were transfected with siRNA targeting TMEM16E for 72 h before determining endogenous TMEM16E mRNA level by quantitative PCR. **C.** HUVECs were transfected with siRNA targeting TMEM16F for 72 h. TMEM16F protein was determined by SDS-PAGE and immunoblotting with anti-TMEM16F antibody. **D.** Primary HUVECs or HUVECs stably expressing TMEM16E-V5 were transfected with indicated siRNA for 72 h prior to determination of TMEM16E and TMEM16F protein by SDS-PAGE and immunoblotting with anti-TMEM16F and anti-V5 antibody. **E.** HUVECs were transfected with indicated siRNA for 72 h and cell viability was determined by XTT assay. #1 and #2 denote independent siRNA sequences. TF denotes siRNA targeting tissue factor.

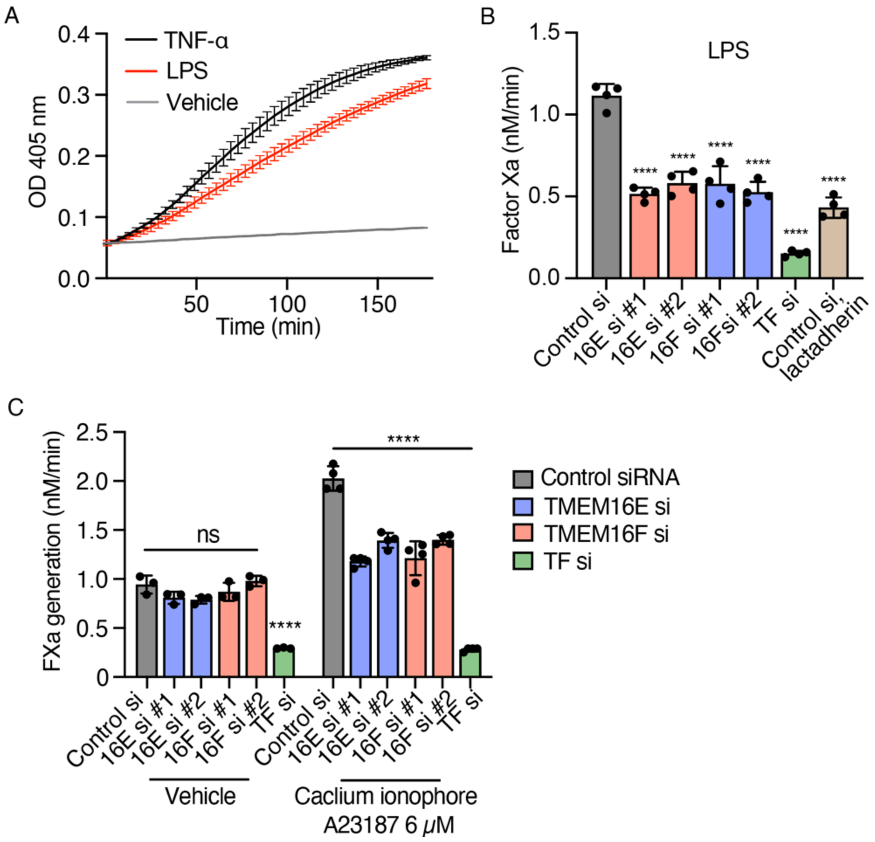

Supplemental Figure 3. TMEM16E and TMEM16F regulate endothelial cell (EC) procoagulant activity. A. Primary HUVECs were stimulated with TNF-α (10 ng/mL), LPS complex (LPS [100 ng/mL], LBP [10 ng/mL], and sCD14 [100 ng/mL]) or vehicle control for 3.5 h and assayed for their ability to support factor VIIa-catalyzed activation of factor X. **B.** HUVECs were transfected with individual siRNAs for 72 h and assayed for their ability to support factor Xa generation following stimulation with LPS complex for 3.5 h. **C.** An Ea.hy926 cell line stably expressing TF was transfected with siRNAs targeting TMEM16E, TMEM16F, or TF for 72 h. Cells were treated with Ca^{2+} ionophore A23187 (6 μM) for 20 min and assayed for their ability to support factor VIIa-catalyzed activation of factor X. 16E, 16F and TF denote siRNA targeting TMEM16E, TMEM16F, and tissue factor, respectively. #1 and #2 denote independent siRNA sequences. Error bars indicate mean ± SEM (**A**) or mean ± SD (**B** and **C**), ANOVA with Tukey's posttest, ****$p<0.0001$.

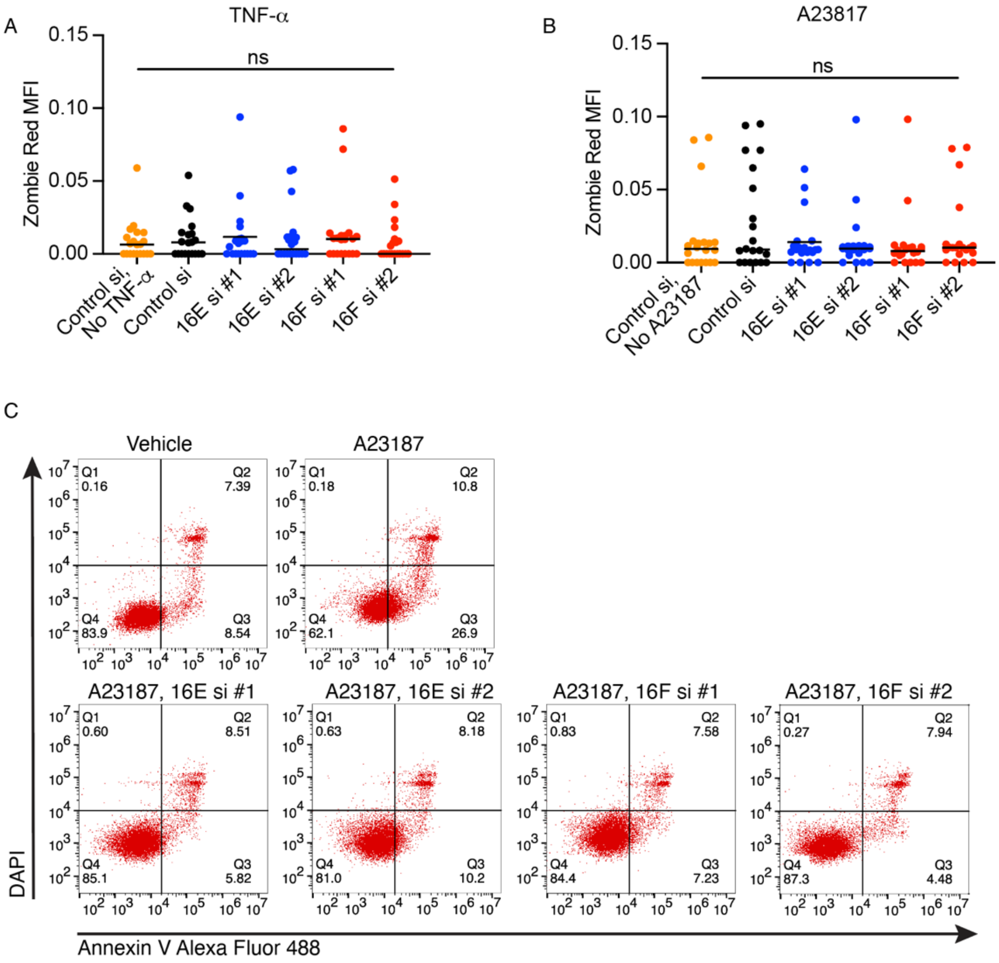

Supplemental Figure 4. TMEM16E and TMEM16F are required for PS externalization on ECs. HUVECs were transfected with indicated siRNAs for 72 h, stimulated with TNF-α (10 ng/mL) for 16 h (**A**) or calcium ionophore A23187 (6 μM) for 20 min (**B**), and stained with annexin V to detect PS externalization and Zombie Red to detect cell death. Each dot represents the total fluorescent area of Zombie Red per image normalized to the number of nuclei present. Note no increase in cell death (Zombie Red positivity) in cells treated with TNF-α or A23187 compared to control. **C**. Representative flow cytometric analysis of PS exposure (annexin V, x-axis) and cell death (DAPI, y-axis). Numbers refer to the percentage of total cells in each quadrant. Q1 represents dead, PS-negative population, Q2 represents dead, PS-positive population, Q3 represents live, PS-positive population, Q4 represents live, PS-negative population.

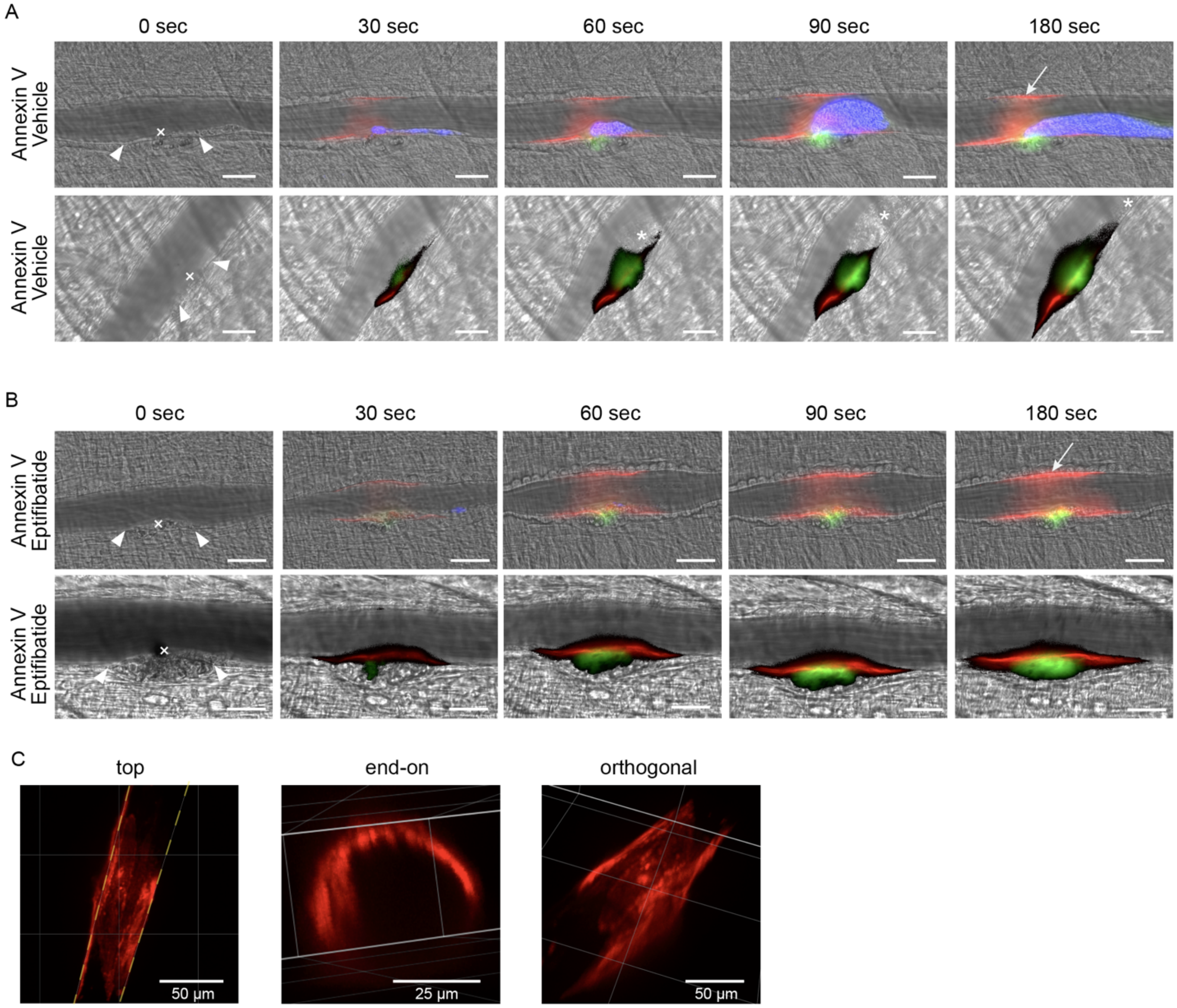

Supplemental Figure 5. PS externalization during thrombus formation following laser injury. Thrombus formation was monitored for 180 seconds in wild-type mice following laser injury of the cremasteric arteriole (**A**) and additionally in the presence of the platelet aggregation inhibitor eptifibatide (**B**, 10 µg/g of body weight). Representative images at indicated time points of the PS probe annexin V (red, Alexa Fluor 647), platelets (anti-CD42b antibody, blue, Dylight 405), and fibrin (anti-fibrin antibody, green, Dylight 488). Note annexin V positivity on the vessel wall and in the absence of platelet aggregation. Arrowheads denote extent of vessel-wall injury and “X” indicates site of laser ablation. Arrows indicate extension of annexin V binding to the vessel wall opposite of laser ablation. To better visualize annexin V binding, platelet fluorescence is omitted from the bottom images in (**A**). Asterisk (*) indicates the platelet aggregate. **C**. 3-dimensional renderings of Z-stack images of annexin V binding following laser injury. Dotted yellow lines indicate the vessel wall boundaries. Scale bar is 25 µm unless otherwise indicated.

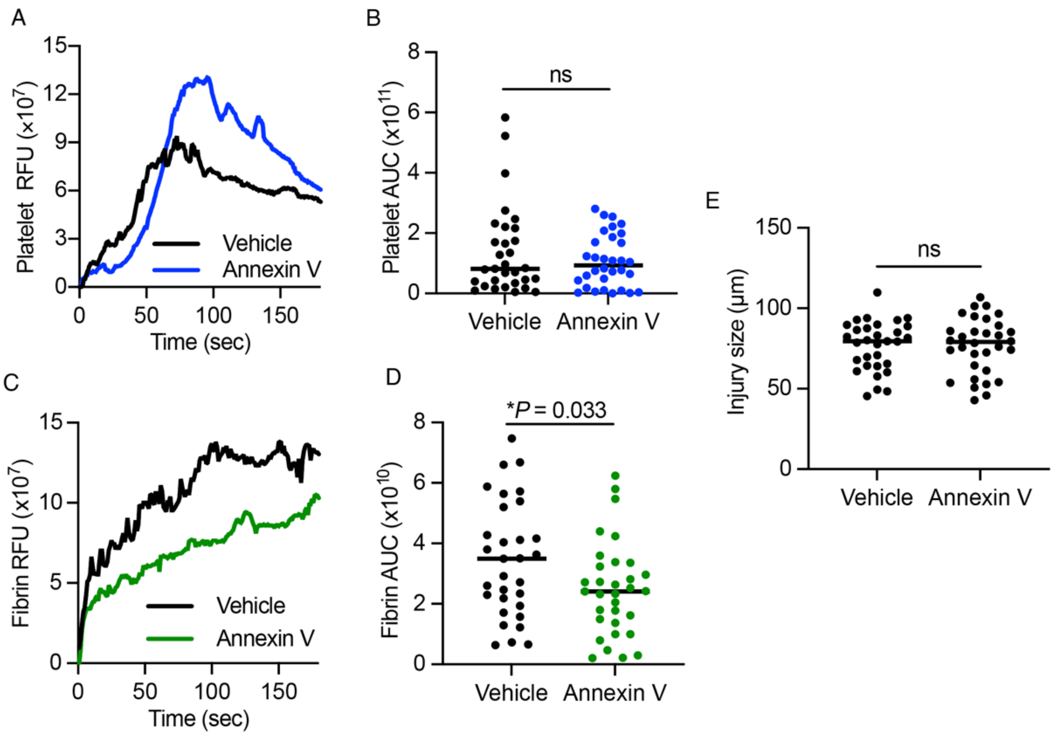

Supplemental Figure 6. Annexin V inhibits thrombosis. Thrombus formation was monitored for 180 seconds in wild-type mice following laser injury of the cremasteric arteriole in the presence or absence of annexin V (0.025 μg/g of body weight). Platelet and fibrin accumulation were monitored by anti-CD42b and anti-fibrin antibody conjugated to Dylight 647 and 488, respectively. Kinetics and magnitude of median integrated relative fluorescent units (RFU) for platelet (**A**) and fibrin (**C**) accumulation are shown following laser injury. The area under the curve (AUC) for fluorescent intensity was determined for platelets (**B**), fibrin (**D**) for each thrombus, analyzed by Mann-Whitney. **E**. Injury sizes associated with the thrombi analyzed above, analyzed by Student's t test.

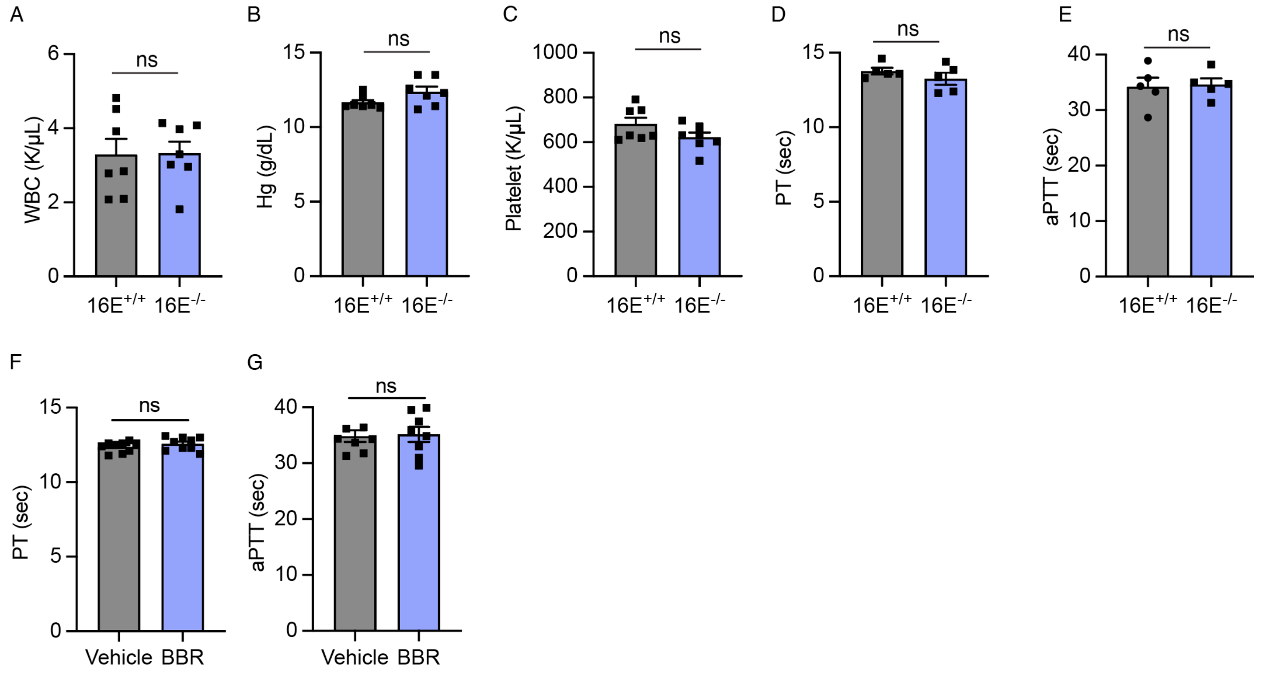

Supplement Figure 7. Blood and coagulation parameters in TMEM16E$^{-/-}$ mice and in wild-type mice following benzbromarone treatment. Blood from TMEM16E$^{-/-}$ (*Ano5*$^{-/-}$) and TMEM16$^{+/+}$ (*Ano5*$^{+/+}$) littermate controls was assessed for white blood cells (WBC, **A**), hemoglobin (Hg, **B**), and platelet counts (**C**). Plasma was assessed for prothrombin time (PT, **D**) and activated partial thromboplastin time (aPTT, **E**). Wild-type C57BL/6J mice were treated with intraperitoneal injection of benzbromarone (5 µg/g of body weight). After 1 h, plasma was assessed for prothrombin time (PT, **F**) and activated partial thromboplastin time (aPTT, **G**). Error bars indicate mean ± SEM, n = 5-8 animals per genotype or treatment.

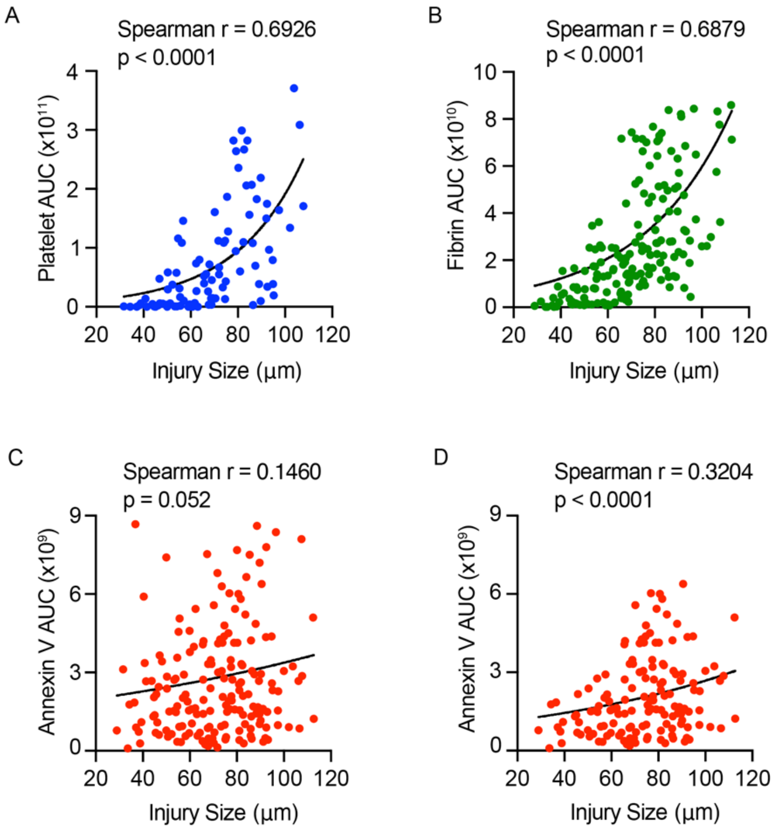

Supplemental Figure 8. Correlation of annexin V binding with vessel wall injury size. Injury size (in µm) following laser ablation plotted against the area under the curve (AUC) for platelets (**A**), fibrin (**B**), and annexin V (**C**) demonstrates a strong Spearman correlation for platelets and fibrin ($p < 0.0001$) but not for annexin V. **D**. Annexin V fluorescence correlation with injury size after removing injuries where annexin V had significant crossover to the vessel wall opposite of laser ablation. Curve fitting was performed with an exponential growth equation.

Supplemental Movie 1. PS externalization and thrombus formation following vascular injury. The following probes were injected into the mouse vasculature: annexin V conjugated to Alexa Fluor 647 to detect PS externalization (red), anti-CD42b antibody conjugated to Dylight 405 to detect platelets (blue), anti-fibrin antibody conjugated to Dylight 488 to detect fibrin (green). Vessel wall injury was induced by laser ablation in a cremasteric arteriole and intravital video microscopy was performed for 180 seconds.

Supplemental Movie 2. PS externalization and fibrin formation in the absence of platelet accumulation. Mice were treated with eptifibatide (10 µg/g body weight). PS, platelets, and fibrin were detected as in Supplemental Movie 1. PS externalization is detected on the vessel wall in the absence of platelet aggregation.

References

1. Higgins SJ et al. Tie2 protects the vasculature against thrombus formation in systemic inflammation. *J. Clin. Invest.* 2018;128(4):1471–1484.

UNCUT GELS (see Figure 2)

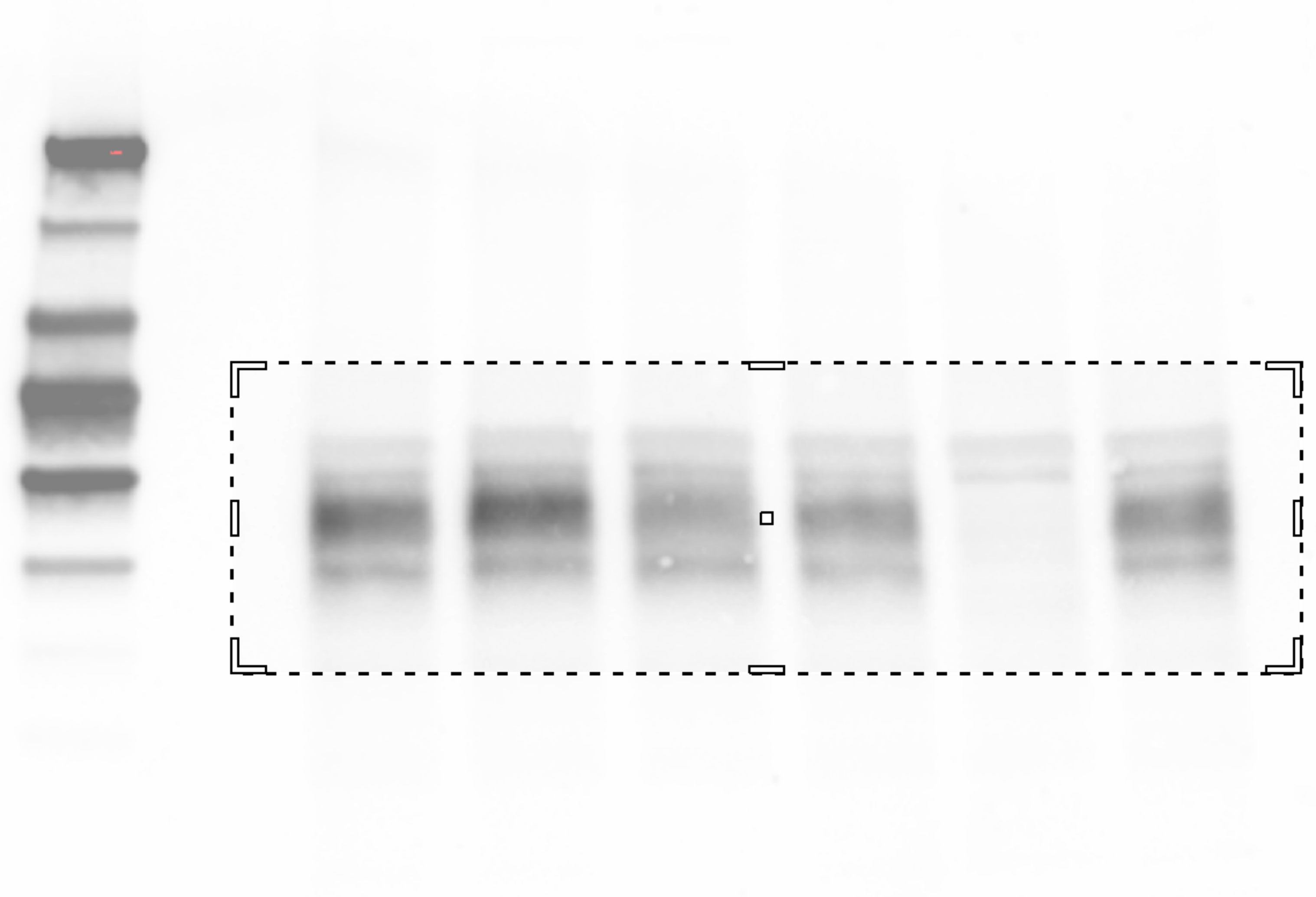

UNCUT GELS (see Figure 2)

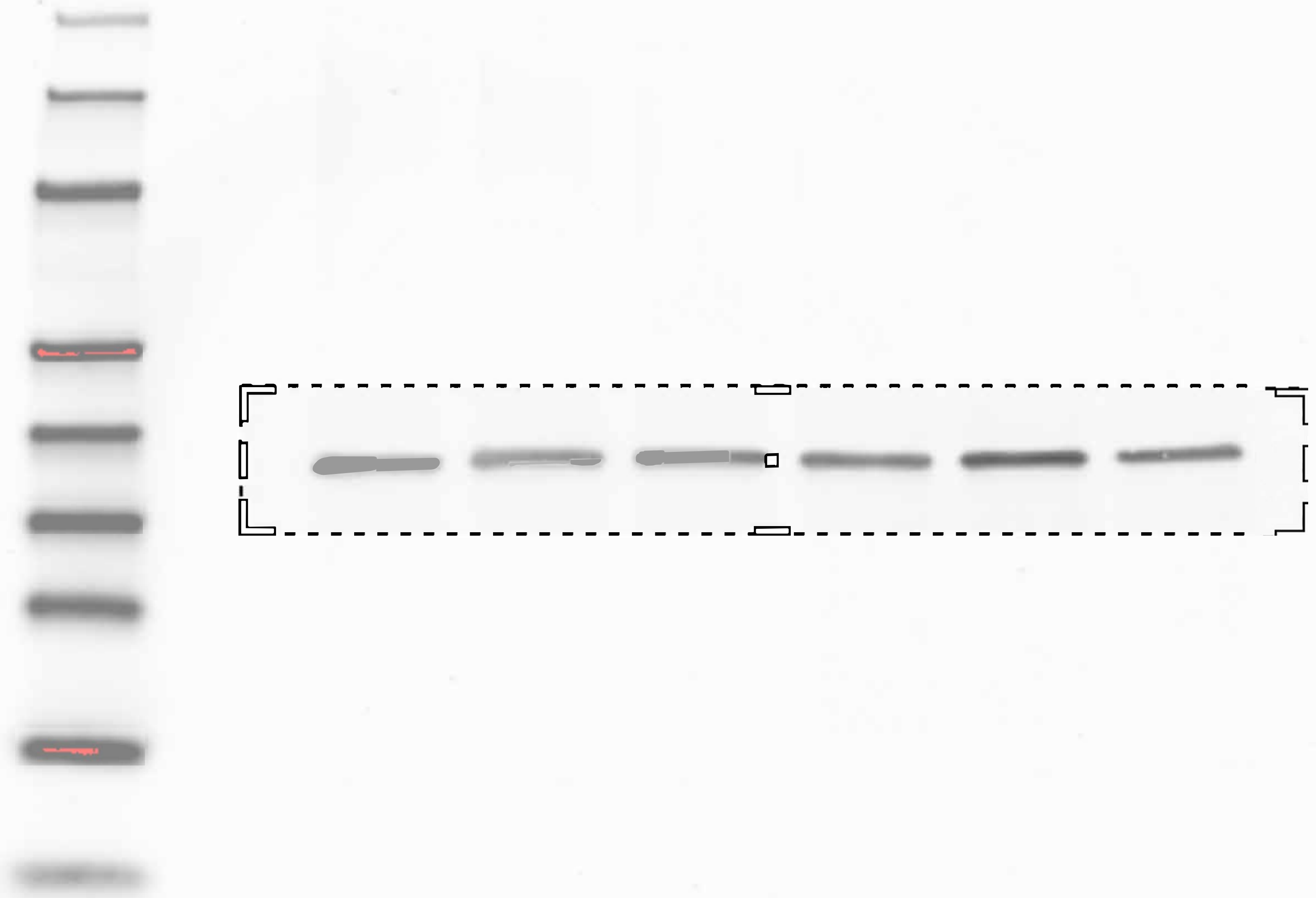

Supplement: Supplemental data [file jci-133-163808-s169.pdf]
